# C9orf72-associated SMCR8 protein binds in the ubiquitin pathway and with proteins linked with neurological disease

**DOI:** 10.1186/s40478-020-00982-x

**Published:** 2020-07-16

**Authors:** John L. Goodier, Alisha O. Soares, Gavin C. Pereira, Lauren R. DeVine, Laura Sanchez, Robert N. Cole, Jose Luis García-Pérez

**Affiliations:** 1 McKusick-Nathans Department of Genetic Medicine, Johns Hopkins University School of Medicine, Baltimore, MD USA; 2 Mass Spectrometry and Proteomics Facility, Department of Biological Chemistry, Johns Hopkins University School of Medicine, Baltimore, MD USA; 3 GENYO. Centre for Genomics and Oncological Research: Pfizer, University of Granada, Andalusian Regional Government, Granada, Spain; 4 MRC Human Genetics Unit, Institute of Genetics and Molecular Medicine (IGMM), University of Edinburgh, Western General Hospital, Edinburgh, UK

**Keywords:** Amyotrophic lateral sclerosis, Autophagy, Biomarker, Mass spectrometry, Proteasome, Stress granules, Ubiquitin

## Abstract

A pathogenic GGGCCC hexanucleotide expansion in the first intron/promoter region of the *C9orf72* gene is the most common mutation associated with amyotrophic lateral sclerosis (ALS). The C9orf72 gene product forms a complex with SMCR8 (Smith-Magenis Syndrome Chromosome Region, Candidate 8) and WDR41 (WD Repeat domain 41) proteins. Recent studies have indicated roles for the complex in autophagy regulation, vesicle trafficking, and immune response in transgenic mice, however a direct connection with ALS etiology remains unclear. With the aim of increasing understanding of the multi-functional C9orf72-SMCR8-WDR41 complex, we determined by mass spectrometry analysis the proteins that directly associate with SMCR8. SMCR8 protein binds many components of the ubiquitin-proteasome system, and we demonstrate its poly-ubiquitination without obvious degradation. Evidence is also presented for localization of endogenous SMCR8 protein to cytoplasmic stress granules. However, in several cell lines we failed to reproduce previous observations that C9orf72 protein enters these granules. SMCR8 protein associates with many products of genes associated with various Mendelian neurological disorders in addition to ALS, implicating SMCR8-containing complexes in a range of neuropathologies. We reinforce previous observations that SMCR8 and C9orf72 protein levels are positively linked, and now show in vivo that SMCR8 protein levels are greatly reduced in brain tissues of C9orf72 gene expansion carrier individuals. While further study is required, these data suggest that SMCR8 protein level might prove a useful biomarker for the *C9orf72* expansion in ALS.

## Introduction

Amyotrophic lateral sclerosis (ALS) is a fatal neurodegenerative disease that afflicts about 1 in 50,000 people each year and involves loss of upper and lower motor neurons [1]. Death typically follows 2 to 3 years after first onset. About 95% of cases are sporadic, while the rest have a family history of the disease. ALS also has overlapping clinical presentations with frontotemporal lobar degeneration (FTLD) and its most common subtype frontotemporal dementia (FTD), a neurological condition affecting the frontal and temporal lobes and marked by cognitive and behavioral impairment [2]. About 20% of ALS patients also exhibit FTLD, and ALS and FTLD have been considered to be part of a continuous disease spectrum [3].

A pathogenic GGGCCC hexanucleotide expansion in the first intron/promoter region of the *C9orf72* gene is the most common mutation associated with both ALS (∼11% of all cases) and FTLD/FTD (∼13%) [3–6]. Three possible non-exclusive mechanisms have been proposed by which the *C9orf72* repeat expansion may cause ALS-FTD: 1) haploinsufficiency and loss of C9orf72 protein function, 2) repeat-associated non-AUG (RAN) translation of the hexanucleotide repeats generating dipeptide repeats that aggregate in toxic neuronal cytoplasmic and nuclear aggregates, and 3) toxic gain-of-function from repeat-containing RNA which forms nuclear foci that sequester hexanucleotode repeat-binding proteins (reviewed in [7–10]). While most studies have focused on increasedtoxicity, accumulating evidence argues that a loss-of-function mechanism may also contribute to neurodegeneration. Consistently, various studies have reported a reduction in *C9orf72* mRNA and/or protein expression in brain and induced pluripotent stem cell (iPSC)-derived neuronal lines of some *C9orf72* ALS (C9ALS) and FTD patients [4–6, 11–25].

A series of studies have shown that the long isoform of human C9orf72 protein forms a complex with SMCR8 (Smith-Magenis Syndrome Chromosome Region, Candidate 8) and WDR41 (WD Repeat domain 41) proteins [22, 26–35]. The *SMCR8* gene is within the deleted region of chromosome 17 associated with Smith-Magenis Syndrome (SMS), a developmental disorder of children involving intellectual disability, distinctive facial features, and behavioral problems, but no reported motor defects [36, 37]. WDR41 is a member of the WD-repeat family of proteins that act as protein-protein or protein-DNA interaction scaffolds for a variety of cellular functions [38]. SNPs within the *WDR41* gene region have been associated with human caudate volume [39].

Bioinformatic analyses first identified both C9orf72 and SMCR8 proteins as having DENN (Differentially Expressed in Normal and Neoplastic cells) domains that are present in guanine nucleotide exchange factors (GEFs) for Rabs, multi-functional small GTPases involved in intracellular membrane trafficking and fusion, vesicle formation and transport, and autophagy [40–42]. The autolysomal-autophagy pathway involves generation of the autophagosome, an organelle surrounded by a double lipid bilayer. Autophagosomes engulf cytoplasmic components, such as protein aggregates, damaged organelles, and foreign pathogens, and fuse with lysosomes to generate autolysosomes that mediate degradation of the cargo. Autophagosomes also fuse with endosomes, forming an intermediate organelle called the amphisome, before fusion with lysosomes. Various studies have linked wild-type C9orf72 protein with proteostasis, showing that, in complex with SMCR8 and WDR41, it bind Rabs and plays roles in autophagy and initiation of autophagosome formation, as well as being linked by function and colocalization to endocytosis and lysosomal and endosomal trafficking ([9, 21–23, 26, 28, 29, 31, 32, 34, 43–48], and Discussion for review). A role in the endo-lysosome pathway has also been shown for the *C. elegans C9orf72* ortholog *alfa-1* [49]. Aoki et al. [46] linked the interaction of C9orf72 and RAB7L1 with regulation of vesicle trafficking, and WDR41 is necessary for recruitment of the C9orf72 complex to lysosomes [35, 50]. Thus, C9orf72 is a regulator of cellular proteostasis.

Additional roles for the C9orf72 complex have also been reported. C9orf72 alters phosporylation of cofilin and activates the small GTPase ADP-ribosylation factor-1/2 (ARF1/2) involved in actin dynamics [51]. Altered C9orf72 protein levels also causes changes in glutamatergic receptor levels, glutamate cycling and endothelin signaling, and excitotoxicity in response to glutamate, as well as widespread transcriptional changes [21, 52–54]. However, the consequences of loss of C9orf72 protein for motor neuron function remain unclear. In vivo, diminished motor function and axonal degeneration of motor neurons have been reported in zebrafish and *C. elegans* depleted of C9orf72 [55, 56]. However, subsequent studies detected no or only mild motor function defects in mice deficient for murine C9orf72 ortholog 3110043O21Rik [45]. On the other hand, in a gain-of-function C9ALS/FTD mouse model, Shao et al. [57] found that 3110043O21Rik haploinsufficiency or loss was associated with increased motor behavior deficits in a dose-dependent manner, while Liang et al. [25] reported that Smcr8 knockout (KO) mice displayed motor behavior defects and axonal swelling. While effects on motor function are uncertain, immune system pathology, spleen and lymph node enlargement, defects in macrophage, myeloid and microglial cell function, altered lysosomal trafficking, and decreased body weight and survival have all been reported for *C9orf72* or *SMCR8* knockout mice [21, 28, 32, 45, 58–65]. Despite these findings, so far no pathogenic loss-of-function coding mutation in *C9orf72*, *SMCR8* or *WDR41* genes has been found [66].

To increase understanding of the diverse functions of the C9orf72-SMCR8-WDR41 complex, we sought to determine by mass spectroscopy (MS) analyses the interactome composition of the SMCR8 component. Notably, we found that the SMCR8 complex includes numerous ubiquitin-related proteins and products of genes associated with numerous Mendelian neurological disorders. MS analyses, co-IP experiments, and association of SMCR8 with cytoplasmic stress granules (SGs) in cultured cells support a link between SMCR8 and the ubiquitin pathway. Furthermore, we reinforce previous observations that SMCR8 and C9orf72 protein levels are positively linked, now showing in vivo that SMCR8 might prove to be a useful biomarker for the C9orf72 expansion mutation in ALS patients.

## Materials and methods

### Plasmid constructs

Ultimate ORF cDNA clones (Invitrogen), with V5-epitope tags and tobacco etch virus (TEV) protease cleavage sites on their N-termini, were recloned by shuttling them from pENTR221 vector into pcDNA3.1/nV5-DEST using Gateway Technology (Invitrogen). Ultimate ORF Clones included AIFM1 (clone identifier IOH61019), BAG5 (IOH26366), C9orf72 (IOH45695), DARS (IOH4209), DCTN1 (IOH42830), DDB1 (IOH13816), DNAJC7 (IOH14566), FKBP5 (IOH13816), G3BP1 (IOH7337), GTF2I (IOH62625), HSPB1 (IOH10530), MAGED2 (IOH3451), PARK7 (IOH3149), PPP2R1A (IOH13670), PSMA7 (IOH5011), PSMC5 (IOH3508), PSMD14 (IOH40112), QARS (IOH3529), RAB1A (IOH2921), RAB7A (IOH40569), RAB11A (IOH13764), RANGAP1 (IOH13287), RO60 (IOH22411), RNF40 (IOH6599), RUVBL2 (IOH3426), SLC25A6 (IOH5815), SQSTM1 (IOH5103), STIP1 (IOH5061), STUB1 (IOH56981), SUGT1 (IOH2955), and TBK1 (IOH21006).

Clones purchased or obtained as gifts included FLAG-UBR5 (pCMV-Tag2B EDD; Addgene plasmid #37188 [67]), pcDNA5 FRT/TO FLAG-SMCR8 (FL-SMCR8), pcDNA5 FRT/TO HA-SMCR8, and pcDNA5 FRT/TO GFP SMCR8 (MRC PPU Reagents and Services, University of Dundee), GFP-(GA)_50_ (L. Petrucelli, Mayo Clinic, Florida, [68]) pRK5-myc-TDP-43 (J. Wang, Johns Hopkins University, [69]), mRFP-UBB (Addgene plasmid #11935, [70]), HA-UBB (V. Dawson, Johns Hopkins University, [71]), and pDest51-USP9X-V5 (R. Hughes, Buck Institute for Research on Aging, [72]). FL-UBB was generated using Ultimate ORF Clone IOH56688 and modified Gateway vector pEZYflag (Addgene plasmid #18700, Y.-Z. Zhang). C9orf72-FL, RO60-FL, SMCR8-V5, and WDR41-FL constructs were generated by PCR-amplification of Ultimate ORF cDNA clones, using a primer with AAG linker and C-terminal FLAG- or V5-tag, and cloning of the products in pcDNA6/myc-His B (pcDNA6, Invitrogen).

### Cell line and tissue samples

Human embryonal carcinoma 2102Ep (a gift from P.K. Andrews, University of Sheffield, UK) and nTERA2D1 cells, human cervical cancer HeLa-JVM cells [73], human embryonic kidney (HEK) 293T cells (ATCC), human neuroblastoma SH-SY-5Y cells (ATCC CRL-2266), and mouse neuroblastoma Neuro2A cells (a gift from D. Hackam, JHU) were grown in Dulbecco’s modified Eagle’s Medium. Mouse hybrid spinal cord neuron/neuroblastoma NSC-34 cells (a gift from D. Griffen, JHU) and human neuroblastoma SK-N-SH cells (a parental line of SH-SY-5Y cells; gifts of D. Valle, JHU) were grown in Eagle’s Minimum Essential Medium. Medium was supplemented with 10% FBS (Hyclone), GlutaMax, and Pen-Strep (Invitrogen). Plasmid transfections used FuGENE HD (Promega) reagent. As necessary, cells were treated with 20 μM MG-132 proteasome inhibitor (Cell Signaling) for about 20 h to inhibit protein degradation, or with 0.25 mM sodium arsenite (NaAsO_2_) or 3 mM dithiothreitol (DTT, Sigma) for 80 min and 2 h, respectively, to induce cytoplasmic SGs.

Post-mortem ALS spinal cord and unaffected control brain motor cortex tissues were obtained from Drs. J. Ravits and R. Batra of the Department of Neurosciences, University of California San Diego School of Medicine [74], and C9ALS and unaffected control samples were from the Target ALS Multicenter Postmortem Tissue Core (Table S4). C9ALS samples had been confirmed for the C9orf72 expansion using repeat-primed PCR (RP-PCR) and Illumina Expansion Hunter (M. Harms, Columbia University, pers. comm.). Frozen spinal cord tissues were obtained from the University of Maryland Brain and Tissue Bank of the NIH NeuroBioBank. Frozen C9ALS and unaffected control cerebrospinal fluid (CSF) samples were from the Northeast ALS Consortium (NEALS). CSF was resuspended directly in 3X SDS loading buffer or first concentrated by tricholoracetic acid precipitation, and then analyzed by Western blotting using α-SMCR8 antibodies. Up to 20 μg of CSF total protein were loaded per well.

### Protein isolation and immunoprecipitation

For MS sequence determination, HEK 293T cells in T_75_ flasks were transfected using FuGENE HD (Promega) with 15 μg of FL-SMCR8, C9orf72-FL, or pcDNA6/myc-His B empty vector and expanded for approximately 45 h, followed by whole cell lysate preparation by sonication using a Diagenode Bioruptor. IP and sample recovery were as previously described [75, 76]. Treatment of samples with 25 μg/ml DNase-free RNase (Roche) and 25 μg/ml RNaseA (Qiagen) was conducted in the absence of RNase inhibitors.

For other protein extracts, tissues or cells were lysed in RIPA buffer (Sigma) with Mammalian Protease Inhibitor Cocktail and phenylmethanesulfonyl fluoride (Sigma) and homogenized by Diagenode Bioruptor. For tissues, 2 mm ziconium silicate beads (Next Advance, Inc.) were added to the tubes. Supernatants were recovered by centrifugation at 11K rpm at 4 °C for 15 min and resuspended in 3X SDS loading buffer.

For each interaction co-IP (Fig. 2), extracts from approximately 5 × 10^6^ 293T cells in T_75_ flasks transfected with tagged SMCR8 and test protein constructs were prepared in 700 μl of lysis buffer (160 mM NaCl, 50 mM Tris, 1 mM EDTA, 0.25% NP40) containing protease and phosphatase inhibitors (Sigma), and RNasin (Qiagen) and vanadyl ribonucleoside complexes (Sigma) as required, and immunoprecipitated as previously described [75, 76].

### Antibody analyses

Commercial antibodies included mouse (ms) α-V5-tag (Invitrogen), rabbit (rb) α-DYKDDDDK-tag (α-FLAG) (D6W5B), ms α-HA-tag (6E2), rb α-Myc-tag (71D10), rb α-HSP90, and ms α-Ubiquitin (UBB) (P4D1) (Cell Signaling Technology), rb α-4E-T, rb α-C9orf72 (S-14), goat (gt) α-eIF3η (N-20), ms α-p70 S6 kinase (which recognizes HEDLS/EDC4 [77]), gt α-TIA1 (C-20), and rb α-WDR41 (S-12) (Santa Cruz Biotechnology), rb α-SMCR8 (ab186504, epitope region 841–890) and rb α-SMCR8 (ab202283, epitope region 600–650) (Abcam), rb α-C9orf72 (22637–1-AP), rb α-SMCR8 (21125–1-AP, epitope region 588–937), and rb α-WDR41 (26817–1-AP) (Proteintech), and rb α-SMCR8 (A304-694A, epitope region 600–650) (Bethyl). Monoclonal ms α-4H1-ORF1 was kindly provided by Dr. K. Burns (JHU, USA, also Millipore MABC1152, [78]), rb α-C9-L and rb α-C9-S antibodies by Dr. J. Robertson (U. Toronto, Canada, [19, 79]), and α-GW182 human serum (IC-6) by Dr. M. Fritzler (U. Calgary, [80]). Donkey Cy3-, DyLight 488-, or DyLight 549-conjugated, and HRP-conjugated secondary antibodies were from Jackson ImmunoResearch Laboratories.

Western blotting, immunofluorescence (IF) microscopy, and fluorescent in situ hybridization (FISH) were performed as described [81, 82]. All Western blotting used NuPAGE 4–12% Bis-Tris gels and MOPS buffer, except brain tissue analyses of Fig. 5b,c which used NuPAGE 3–8% Tris-acetate gels (Thermo Fisher). Detection used Supersignal West Pico or West Pico Plus Chemiluminescent Substrate (Thermo Fisher) with ECL Hyperfilm (MilliporeSigma).

Immunostained cells were examined using a Nikon Eclipse Ti-A1 confocal microscope with NIS-Elements AR software.

### MS sequencing and data analyses

MS sequencing and database analyses was performed by the Johns Hopkins Mass Spectrometry and Proteomics Facility as previously described [75, 76]. Peptide sequences were identified using Proteome Discoverer and Mascot software (Matrix Science) to search the NCBInr 167 database, including gly-gly modifiation on lysine as a variable protein modification. False discovery rate (FDR) was set at 1.0. Mascot search result *.dat files were processed in Scaffold (Proteome Software, Inc.) to validate protein and peptide identifications. Exclusion criteria for proteins are described in the Results section.

Functional analyses of gene lists obtained by MS sequencing utilized DAVID v6.7 (Database for Annotation, Visualization and Integrated Discovery [83]). Protein multiple alignments used Clustal Omega 1.2.1 (EMBL-EBI) and the ESPript 3.0 web sever (http://espript.ibcp.fr/ESPript/ESPript/). RNA-Seq datasets from public databases (GEO GSE67196 and NeuroLINCS dbGaP Study phs001231 SRP098831) were analyzed for gene expression using the software package TETranscripts as previously described [84, 85]. Ubiquitination prediction algorithms included UbPred (http://www.ubpred.org, [86]), BDM-PUB (http://bdmpub.biocuckoo.org), and UbiSite (http://csb.cse.yzu.edu.tw/UbiSite/).

## Results

### Confirmation of the SMCR8-C9orf72-WDR41 complex and detection using commercial antibodies

**Fig. 1:**
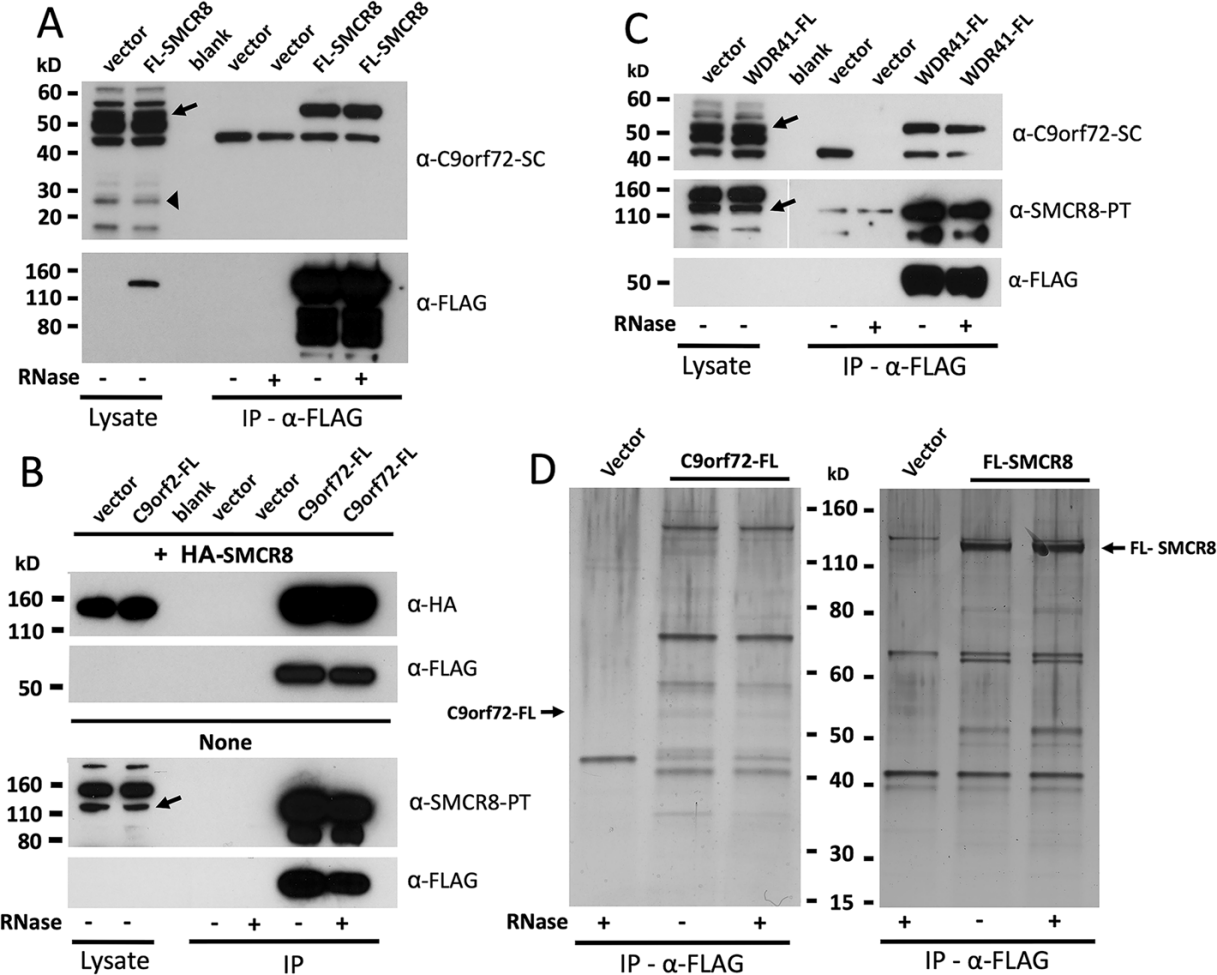
Protein interaction analyses by Western blotting and co-IP of the SMCR8 complex in HEK 293T cells (see Fig. S1 for antibody analyses). **a** Endogenous C9-L (arrow) co-IPs with FLAG-tagged SMCR8. The thick arrowhead marks a band consistent in size with C9-S. **b** FLAG-tagged C9orf72 co-IPs both endogenous and co-transfected HA-tagged SMCR8. **c** FLAG-tagged WDR41 protein co-IPs both endogenous C9orf72 and SMCR8 proteins (indicated by arrows). Tagged C9orf72 and WDR41 proteins of (**b**) and (**c**) are not visible in whole cell lysates at the Western blot film exposure times shown. **d** C9orf72-FL, FL-SMCR8, and empty vector were immunoprecipitated on α-FLAG agarose from transfected 293T whole cell lysates, resolved on a polyacrylamide gel, and silver-stained. IP reactions were in the presence or absence of 50 μg/ml RNases. Complex immunoprecipitate samples were analyzed by MS sequencing. Arrows indicate full-length protein bands. Protein molecular weight markers are those of Novex Sharp Pre-stained Protein Standard (Thermo Fisher Scientific)

Previous studies characterizing the C9orf72 proteome identified a complex consisting of the proteins C9orf72, SMCR8 and WDR41 [22, 26–35]. By means of co-immunoprecipitation (co-IP) and MS sequencing, here we sought to identify proteins that associate with SMCR8. First, we confirmed that C9orf72, SMCR8 and WDR41 proteins formed a stable complex in our overexpression experimental system using human embryonic kidney (HEK) 293T cells. Consistent with previous reports, even in the presence of RNase, epitope-tagged SMCR8 and C9orf72 co-IPed C9orf72 and SMCR8 proteins, respectively (Fig. 1a, b). Furthermore, we confirmed that FLAG-tagged WDR41 interacts with endogenous C9orf72 and SMCR8 (Fig. 1c).

We next assessed the efficacy of several commercial antibodies against these proteins. Human *C9orf72* expresses a 54-kilodalton (kD) long protein isoform (C9-L) and a 25-kD short isoform (C9-S). It has been noted that commercial C9orf72 antibodies often detect additional bands other than C9-L or fail to detect C9-S [17, 19, 23]. Consistent with this observation, the Santa Cruz S-14 antibody (α-C9orf72-SC) detected multiple bands in whole cell lysates as well as products of size consistent with both endogenous C9-L and C9-S; only C9-L co-IPed with tagged SMCR8 or WDR41 (Fig. 1a,c). Similarly, in both cultured cells and brain and spinal cord tissue lysates, the Proteintech 22,637–1-AP antibody (α-C9orf72-PT) marked a major band consistent with C9-L (arrow), plus additional products (Fig. S1A). A small number of non-commercial C9orf72-specific antibodies have also been described [17, 23, 30, 79].

Both mouse and human SMCR8 have two predicted isoforms, the full length 105-kD protein and a C-terminal truncated 87.4-kD isoform generated by alternate splicing [36]. Our search of GenBank revealed additional human *SMRC8* mRNA isoforms potentially encoding 35.9-kD (accession numbers BC001018, BC005067), 75.2-kD (AK296847.1), and 93-kD (BC101116, BC101117) protein products. Tissue-specific transcripts of various sizes have also been experimentally observed for human *SMCR8* [36]. The Proteintech, Bethyl (A304-694A), and Abcam (ab186504 and ab202283) α-SMCR8 antibodies all marked a band consistent in size with full-length SMCR8 (i.e. 105 kD, arrows in Fig. S1B-E), plus additional bands of unknown specificicy, but which could in part relate to the above desribed SMCR8 protein isoforms. The Bethyl and Abcam ab202283 α-SMCR8 antibodies have been used in other studies, and our observations are similar [34, 50, 65, 79]. Interestingly, although expression of full length SMCR8 protein was detected in human brain tissues, none was seen in spinal cord tissue lysates of multiple samples (Fig. S1B-E).

WDR41 has two predicted isoforms of 51.7 and 45.5 kD (Swiss Prot. Q9HAD4–1, Q9HAD4–2). For selected cancer cell lines, both the Santa Cruz (S-12) and Proteintech (26817–1-AP) polyclonal antibodies detected doublet bands consistent in size with these isoforms (Fig. S1F, G). These bands were very faint (Proteintech) or absent (Santa Cruz) from human brain and spinal cord tissue lysates, although bands of larger and smaller sizes were visible by Western blotting of cultured cells.

### SMCR8 interactome contains many central nervous system (CNS) disease proteins

Because of the possible non-specific protein interactions described above, we considered commercial antibodies unsuitable for co-IP interactome studies. Therefore, we exploited a co-IP/MS protocol that we have successfully used in previous studies [75, 76]. We transfected C9-L with C-terminal FLAG (FL)-tag, full-length SMCR8 with N-terminal FLAG-tag, or empty vector control in HEK 293T cells and performed α-FLAG IP from whole cell extracts in the presence or absence of RNase (Fig. 1d). Complex immunoprecipitated samples were analyzed by liquid chromatography tandem MS. After excluding ribosomal proteins and likely contaminants (such as keratins), 340 and 201 proteins having three or more spectra and not detected in vector only control cell lysates were associated with FL-SMCR8 and C9-L-FL, respectively (Tables 1, S1, S2). Furthermore, 71 proteins were found in both proteomes, although it should be noted that C9-L-FL was expressed at significantly lower levels than FL-SMCR8 (Fig. 1d), as previously reported [29]. Tables S1 and S2 also note interacting partners of C9orf72 or SMCR8 proteins reported in previous studies [19, 28, 29, 31, 32, 34, 51, 87, 88]. During the course of our investigations, another MS experiment was published that listed 1532 proteins that co-IPed with HA-tagged SMCR8 from 293T cells [34], and a total of 272 of these (80%) were also present in our dataset (Table S2).

**Table 1:** Summary of the C9orf72 and SMCR8 protein interactomes and selected functional categories

Gene Name	Protein Name	Total # Spectra	
		RNase	
		-	+
**A. C9orf72-FL**			
C9orf72	chromosome 9 open reading frame 72	108	103
**TOP 20 C9orf72-INTERACTING PROTEINS**			
SMCR8	Smith-Magenis syndrome chromosome region, candidate 8	165	217
WDR41	WD repeat domain 41	69	85
FLG2	filaggrin family member 2	33	6
LRPPRC	leucine rich pentatricopeptide repeat containing	23	25
IARS	isoleucyl-tRNA synthetase	21	22
SERPINB3	serpin family B member 3	21	0
CALML5	calmodulin like 5	20	0
HADHA	hydroxyacyl-CoA dehydrogenase/3-ketoacyl-CoA thiolase/enoyl-CoA hydratase, alpha subunit	18	14
CDSN	corneodesmosin	17	8
EEF2	eukaryotic translation elongation factor 2	15	18
FABP5	fatty acid binding protein 5	15	0
COPB1	coatomer protein complex subunit beta 1	14	14
DSC1	desmocollin 1	14	15
U2AF2	U2 small nuclear RNA auxiliary factor 2	14	6
LUC7L3	LUC7 like 3 pre-mRNA splicing factor	13	4
EIF2S3	eukaryotic translation initiation factor 2 subunit gamma	12	10
HNRNPH2	heterogeneous nuclear ribonucleoprotein H2	12	9
KHDRBS1	KH RNA binding domain containing, signal transduction associated 1	12	6
PSMC2	proteasome 26S subunit, ATPase 2	12	25
**B. FL-SMCR8**			
SMCR8	Smith-Magenis syndrome chromosome region, candidate 8	1135	1182
**TOP 25 SMCR8-INTERACTING PROTEINS**			
DNAJA1	DnaJ heat shock protein family member A1	70	76
GTF2I	general transcription factor IIi	55	61
CAD	carbamoyl-phosphate synthetase 2, aspartate transcarbamylase, and dihydroorotase	62	48
ATP1A1	ATPase Na+/K+ transporting subunit alpha 1	38	46
EPRS	glutamyl-prolyl-tRNA synthetase	36	40
MAGED1	MAGE family member D1	26	33
WDR41	WD repeat domain 41	32	33
DNAJC7	DnaJ heat shock protein family member C7	30	32
SLC25A3	solute carrier family 25 member 3	22	32
STUB1	STIP1 homology and U-box containing protein 1	30	31
GCN1	GCN1, eIF2 alpha kinase activator homolog	37	30
DDB1	damage specific DNA binding protein 1	31	29
DYNC1H1	dynein cytoplasmic 1 heavy chain 1	38	29
CHD4	chromodomain helicase DNA binding protein 4	13	28
SLC25A11	solute carrier family 25 member 11	14	28
RANBP2	RAN binding protein 2	29	25
RUVBL2	RuvB like AAA ATPase 2	30	25
ATP2A2	ATPase sarcoplasmic/endoplasmic reticulum Ca2+ transporting 2	19	22
DNAJA3	DnaJ heat shock protein family member A3	19	22
IARS	isoleucyl-tRNA synthetase	18	22
STIP1	stress induced phosphoprotein 1	37	22
COPA	coatomer protein complex subunit alpha	23	20
HUWE1	HECT, UBA and WWE domain containing 1, E3 ubiquitin protein ligase	29	20
AIFM1	apoptosis inducing factor, mitochondria associated 1	18	18
C9orf72	chromosome 9 open reading frame 72	15	18
**AMINOACYL-tRNA SYNTHESIS**			
EPRS	glutamyl-prolyl-tRNA synthetase	40	13
IARS	isoleucyl-tRNA synthetase	18	22
DARS	aspartyl-tRNA synthetase	11	8
MARS	methionyl-tRNA synthetase	10	6
RARS	arginyl-tRNA synthetase	9	8
LARS	leucyl-tRNA synthetase	9	14
QARS	glutaminyl-tRNA synthetase	8	7
TARS2	threonyl-tRNA synthetase 2, mitochondrial (putative)	6	4
TARS	threonyl-tRNA synthetase	4	0
FARSA	phenylalanyl-tRNA synthetase alpha subunit	0	6
**CELL CYCLE**			
SMC3	structural maintenance of chromosomes 3	12	8
CDK4	cyclin dependent kinase 4	5	0
PCNA	proliferating cell nuclear antigen	5	4
MCM7	minichromosome maintenance complex component 7	4	6
YWHAZ	tyrosine 3-monooxygenase/tryptophan 5-monooxygenase activation protein zeta	4	9
RAD21	RAD21 cohesin complex component	3	0
CDC7	cell division cycle 7	3	0
PLK1	polo like kinase 1	3	9
PPP6C	protein phosphatase 6 catalytic subunit	3	0
CDKN2A	cyclin dependent kinase inhibitor 2A	0	3
MCM2	minichromosome maintenance complex component 2	0	4
MCM5	minichromosome maintenance complex component 5	0	4
SMC1A	structural maintenance of chromosomes 1A	0	4
**HEAT SHOCK PROTEINS / CHAPERONES**			
DNAJA1	DnaJ heat shock protein family member A1	70	76
DNAJC7	DnaJ heat shock protein family member C7	30	32
DNAJA3	DnaJ heat shock protein family member A3	19	22
DNAJA3	DnaJ heat shock protein family member A3	19	22
HSPH1	heat shock protein family H member 1	19	8
BAG5	BCL2 associated athanogene 5	18	11
DNAJA2	DnaJ heat shock protein family member A2	15	16
BAG2	BCL2 associated athanogene 2	11	16
BAG6	BCL2 associated athanogene 6	11	8
SERPINH1	serpin family H member 1	11	0
HSPB1	heat shock protein family B member 1	10	9
MDN1	midasin AAA ATPase 1	10	14
FKBP8	FK506 binding protein 8	8	0
DNAJB6	DnaJ heat shock protein family member B6	6	4
PFDN2	prefoldin subunit 2	5	4
DNAJB1	DnaJ heat shock protein family member B1	4	0
HSPA4	heat shock protein family A member 4	4	3
HSPA4	heat shock protein family A member 4	4	3
RBBP7	RB binding protein 7, chromatin remodeling factor	3	4
SERPINB3	serpin family B member 3	3	0
BAG3	BCL2 associated athanogene 3	2	3
UNC45A	unc-45 myosin chaperone A	2	5
DNAJB11	DnaJ heat shock protein family member B11	0	6
DNAJC10	DnaJ heat shock protein family member C10	0	5
PARK7	Parkinsonism associated deglycase	0	4
**MISMATCH REPAIR**			
RFC3	replication factor C subunit 3	5	0
SSBP1	single stranded DNA binding protein 1	5	0
MSH6	mutS homolog 6(MSH6)	5	5
POLD3	DNA polymerase delta 3, accessory subunit	2	3
PCNA	proliferating cell nuclear antigen	0	4
POLD1	DNA polymerase delta 1, catalytic subunit	0	3
**PHAGOSOME/AUTOPHAGY**			
DYNC1H1	dynein cytoplasmic 1 heavy chain 1	38	29
SEC16A	SEC16 homolog A, endoplasmic reticulum export factor	14	12
TUBB4A	tubulin beta 4A class IVa	11	10
IRS4	insulin receptor substrate 4	10	13
TUBB3	tubulin beta 3 class III	9	13
TUBB6	tubulin beta 6 class V	9	18
PPP2R1A	protein phosphatase 2 scaffold subunit Aalpha	7	5
CDK5	cyclin dependent kinase 5	7	0
SEC61B	Sec61 translocon beta subunit	6	0
HLA-A	major histocompatibility complex, class I, A	5	5
ANKRD28	aminoadipate-semialdehyde dehydrogenase-phosphopantetheinyl transferase	5	5
RAB1B	RAB1B, member RAS oncogene family	5	0
SLC3A2	solute carrier family 3 member 2	4	8
SEC24C	SEC24 homolog C, COPII coat complex component	4	3
TFRC	transferrin receptor	4	0
AP3M1	adaptor related proteincomplex 3 Mu 1 subunit	3	0
PPP6C	protein phosphatase 6 catalytic subunit	3	0
BAG3	BCL2 associated athanogene 3	2	3
WDR5	WD repeat domain 5	0	5
YME1L1	YME1 like 1 ATPase	0	4
KEAP1	kelch like ECH associated protein 1	0	4
DYNC1I2	dynein cytoplasmic 1 intermediate chain 2	0	4
CDKN2A	cyclin dependent kinase inhibitor 2A	0	3
HLA-B	major histocompatibility complex, class I, B	0	3
**PROTEIN PROCESSING IN THE ENDOPLASMIC RETICULUM**			
DNAJA1	DnaJ heat shock protein family (Hsp40) member A1	70	76
STUB1	STIP1 homology and U-box containing protein 1	30	31
HSPH1	heat shock protein family H (Hsp110) member 1	19	8
DNAJA2	DnaJ heat shock protein family (Hsp40) member A2	15	16
BAG2	BCL2 associated athanogene 2	11	16
SEC61B	Sec61 translocon beta subunit	6	0
RPN1	ribophorin I	5	3
TXNDC5	thioredoxin domain containing 5	5	2
DNAJB1	DnaJ heat shock protein family (Hsp40) member B1	4	0
SEC24C	SEC24 homolog C, COPII coat complex component	4	3
DDOST	dolichyl-diphosphooligosaccharide--protein glycosyltransferase 48 kD subunit	3	4
SSR4	signal sequence receptor subunit 4	2	3
DNAJB11	DnaJ heat shock protein family (Hsp40) member B11	0	6
DNAJC10	DnaJ heat shock protein family (Hsp40) member C10	0	5
RRBP1	ribosome binding protein 1	0	3
**PROTEOSOME**			
PSMC2	proteasome 26S subunit, ATPase 2	22	18
PSMD2	proteasome 26S subunit, non-ATPase 2	18	10
PSMA7	proteasome subunit alpha 7	17	16
PSMD11	proteasome 26S subunit, non-ATPase 11	14	9
PSMC3	proteasome 26S subunit, ATPase 3	12	9
PSMD3	proteasome 26S subunit, non-ATPase 3	12	8
PSMA5	proteasome subunit alpha 5	11	6
PSMC5	proteasome 26S Subunit, ATPase 5	10	10
PSME3	proteasome activator subunit 3	10	11
PSMA6	proteasome subunit alpha 6	9	0
PSMD6	proteasome 26S subunit, non-ATPase 6	9	7
PSMA3	proteasome subunit alpha 3	8	3
PSMD1	proteasome 26S subunit, non-ATPase 1	8	8
PSMA1	proteasome subunit alpha 1	7	7
PSMB1	proteasome subunit beta 1	7	7
PSMB5	proteasome subunit beta 5	6	6
PSMC1	proteasome 26S subunit, ATPase 1	6	6
PSMB2	proteasome subunit beta 2	5	5
PSMB6	proteasome subunit beta 6	5	5
PSMC4	proteasome 26S subunit, ATPase 4	5	0
PSMC6	proteasome 26S subunit, ATPase 6	5	6
PSMD12	proteasome 26S subunit, non-ATPase 12	4	2
PSMD7	proteasome 26S subunit, non-ATPase 7	3	0
PSMD13	proteasome 26S subunit, non-ATPase 13	0	3
PSMD14	proteasome 26S subunit, non-ATPase 14	0	3
PSMD8	proteasome 26S subunit, non-ATPase 8	0	6
**RNA TRANSPORT**			
RANBP2	RAN binding protein 2	29	25
XPO1	exportin 1	13	13
NUP93	nucleoporin 93	11	10
NUP133	nucleoporin 133	10	15
THOC2	THO complex 2	9	6
RANGAP1	Ran GTPase activating protein 1	8	11
KPNB1	karyopherin subunit beta 1	7	5
KPNA2	karyopherin subunit alpha 2	6	4
EIF4A1	eukaryotic translation initiation factor 4A1	5	4
EIF4G1	eukaryotic translation initiation factor 4 gamma 1	5	4
NUP155	nucleoporin 155	4	6
NUP210	nucleoporin 210	4	0
EIF3F	eukaryotic translation initiation factor 3 subunit F	3	3
FXR2	FMR1 autosomal homolog 2	3	0
NUP160	nucleoporin 160	3	4
NUP205	nucleoporin 205	3	4
EIF3E	eukaryotic translation initiation factor 3 subunit E	2	4
FMR1	fragile X mental retardation 1	2	0
NUP98	nucleoporin 98	2	5
NUP153	nucleoporin 153	0	5
THOC3	THO complex 3	0	4
XPOT	exportin for tRNA	0	4
**UBIQUITINATION / SUMOYLATION**			
STUB1	STIP1 homology and U-box containing protein 1	30	31
HUWE1	HECT, UBA and WWE domain containing 1, E3 ubiquitin ligase	29	20
RANBP2	RAN binding protein 2, E3 SUMO ligase	29	25
RNF40	ring finger protein 40, E3 ubiquitin ligase	4	10
USP7	ubiquitin specific peptidase 7	4	9
USP9X	ubiquitin specific peptidase 9, X-linked	4	5
RNF2	ring finger protein 2, , E3 ubiquitin protein ligase	3	0
UBR5	ubiquitin protein ligase E3 component n-recognin 5	3	12
USP15	ubiquitin specific peptidase 15	3	0
SUMO3	small ubiquitin-like modifier 3	0	3
MKRN2	makorin ring finger protein 2, E3 ubiquitin ligase	0	3

**Fig. 2:**
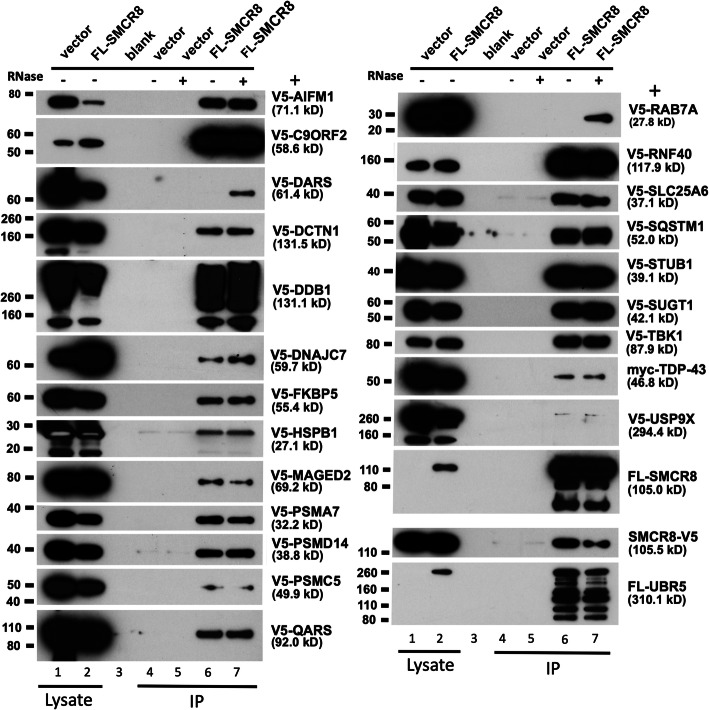
Confirmation of proteins in the SMCR8 complex. Selected proteins detected in the SMCR8 interactome by MS sequencing were tagged and coexpressed with SMCR8 in HEK 293T cells. Most were found to specifically co-IP on α-FLAG agarose with FL-SMCR8 but not empty vector. Approximately 1% of the input lysate (lanes 1, 2) and 30% of the immunoprecipitate (lanes 4–7) were loaded on gels. IP reactions were in the presence or absence of 50 μg/ml RNases. Also included is a panel representative of tagged FL-SMCR8 protein present in the input and IP fractions (detected by α-FLAG antibody) and showing that RNase treatment did not affect SMCR8 immunoprecipitation (lower right). Test proteins were detected by α-V5 antibody, except FL-UBR5, which was detected by α-FLAG antibody (bottom right). The molecular weight of each test protein, including its epitope tag, is shown in brackets. Protein molecular weight markers are those of Novex Sharp Pre-stained Protein Standard (Thermo Fisher)

To further confirm the effectiveness of our MS analyses, we next analyzed some of the interactors identified. To do that, a subset of cDNAs identified from the SMCR8 proteome were cloned with an N-terminal V5-TEV (tobacco etch virus)-epitope tag or were obtained as gifts. Notably, following cotransfection in 293T cells, 73% (22/30) of proteins tested directly co-IPed with FL-SMCR8 on α-FLAG agarose, further confirming the efficiency of our protocol (Fig. 2). In almost all cases, interactions were resistant to RNase digestion. Some proteins bound non-specifically to the agarose (BAG5, PPP2R1A, RUVBL2) or failed to bind FL-SMCR8 (G3BP1, GTF2I, RAB1A, RANGAP1, STIP1). It is possible some of these latter proteins are only able to bind SMCR8 when in complex with over-expressed C9orf72 and/or WDR41.

Several studies have proposed a role for C9orf72 in the regulation of autophagy by Rab GTPases, although with disagreement concerning which of the many Rab family members binds the C9orf72/SMCR8/WDR41 complex. Farg et al. [43] first reported C9orf72 to interact with RAB1, RAB5, RAB7 and RAB11. Webster et al. [22] confirmed that C9orf72 associates with GTP-bound RAB1A and the ULK1 complex, and it has been demonstrated that C9orf72 in complex with SMCR8 and WDR41 is a GEF for RAB8A, RAB11A, and RAB39B, and that its loss perturbs autophagy in neurons [27, 29, 31, 89]. We detected only RAB1B in our SMCR8 and C9orf72 interactomes (Tables S1, S2), but failed to confirm binding of V5-tagged RAB1A, a paralog highly similar in sequence to RAB1A, with SMCR8 in direct co-IP experiments. However, we also tested and confirmed weak binding of V5-RAB7A with overexpressed SMCR8 (Fig. 2) and C9orf72 (not shown), but only in the presence of RNase.

Significantly, when we queried the OMIM (Online Mendelian Inheritence in Man) database (https://omim.org/), we found that 65 (19%) of our putative SMCR8-interacting proteins are associated with neurodegenerative and neurological genetic disorders (Table 2). These include 8 proteins linked with ALS and/or FTD, 14 with other neurodegenerative diseases (including 4 associated with spinocerebellar ataxias), 7 with Charcot-Marie Tooth disease, 5 with hypomyelinating leukodystrophy, and 13 with mental retardation. Thus, SMCR8 may recruit some of these proteins to its complex with C9orf72 and WDR41, predicting roles for the complex in central nervous system (CNS) disorders.

**Table 2:** Proteins of the SMCR8 interactome associated with CNS disease according to the Online Mendelian Inheritance in Man (OMIM) database

GENE NAME	DISEASE NAME	SYMBOL	OMIM #
**ALS/FTD**			
ATXN2	Amyotrophic lateral sclerosis 13	ALS13	183,090
C9orf72	Frontotemporal dementia and/or amyotrophic lateral sclerosis 1	FTDALS1	105,550
DCTN1	Amyotrophic lateral sclerosis	ALS	105,400
FIG4	Amyotrophic lateral sclerosis 11	ALS11	612,577
FUS	Amyotrophic lateral sclerosis 6, with or without frontotemporal dementia	ALS6	608,030
MATR3	Amyotrophic lateral sclerosis 21	ALS21	606,070
SQSTM1	Frontotemporal dementia and/or amyotrophic lateral sclerosis 3	FTDALS3	616,437
TARDBP	Amyotrophic lateral sclerosis 10	ALS10	612,069
**OTHER NEURODEGENERATION DISEASES**			
AIFM1	Combined oxidative phosphorylation deficiency 6	COXPD6	300,816
ATXN2	Spinocerebellar ataxia 2	SCA2	183,090
CTSD	Ceroid lipofuscinosis, neuronal, 10	CLN10	610,127
DYNC1H1	Spinal muscular atrophy, lower extremity-predominant 1, autosomal dominant	SMALED1	158,600
EIF4G1	Parkinson disease 18	PARK18	614,251
NOP56	Spinocerebellar ataxia 36	SCA36	614,153
PARK7	Parkinson disease 7	PARK7	606,324
PCNA	Ataxia-telangiectasia-like disorder 2	ATLD2	615,919
PUM1	Spinocerebellar ataxia 47	SCA47	617,931
QARS	Microcephaly, progressive, with seizures and cerebral and cerebellar atrophy	MSCCA	615,760
RARS	Leukodystrophy, hypomyelinating, 9	HLD9	616,140
SPG20	Spastic paraplegia 20, autosomal recessive	SPG20	275,900
STUB1	Spinocerebellar ataxia, autosomal recessive, 16	SCAR16	615,768
WNK1	Neuropathy, hereditary sensory and autonomic, 2A	HSAN2A	201,300
**OTHER NEUROLOGICAL CONDITIONS**			
ADAR	Aicardi-Goutieres syndrome 6	AGS6	615,010
ADNP	Helsmoortel-van der Aa syndrome	HVDAS	615,873
AIFM1	Cowchock syndrome	COWCK	310,490
AIMP1	Leukodystrophy, hypomyelinating, 3	HLD3	260,600
AIMP2	Leukodystrophy, hypomyelinating, 17	HLD17	618,006
ALDH18A1	Spastic paraplegia 9A, autosomal dominant	SPG9A	601,162
ALDH18A1	Spastic paraplegia 9B, autosomal recessive	SPG9B	616,586
ALDH3A2	Sjogren-Larsson syndrome	SLS	270,200
ARHGEF2	Neurodevelopmental disorder with midbrain and hindbrain malformations	NEDMHM	617,523
ATAD3A	Harel-Yoon syndrome	ATAD3A	612,316
ATP1A1	Charcot-Marie-Tooth disease, axonal, type 2DD	CMT2DD	618,036
CAD	Epileptic encephalopathy, early infantile, 50	AR	616,457
CDK5	Lissencephaly 7, with cerebellar hypoplasia	LIS7	616,342
CLTC	Mental retardation, autosomal dominant 56	MRD56	617,854
COPB2	Microcephaly 19, primary, autosomal recessive	MCPH19	617,800
DARS	Hypomyelination with brainstem and spinal cord involvement and leg spasticity	HBSL	615,281
DCTN1	Neuronopathy, distal hereditary motor, 7B	HMN7B	607,641
DCTN1	Perry syndrome	PERRYS	168,605
DOCK7	Epileptic encephalopathy, early infantile, 23	AR	615,859
DYNC1H1	Charcot-Marie-Tooth disease 2O	CMT2O	614,228
DYNC1H1	Mental retardation, autosomal dominant 13	MRD13	614,563
EPRS	Leukodystrophy, hypomyelinating, 15	HLD15	617,951
FIG4	Polymicrogyria, bilateral temporooccipital	BTOP	612,691
FIG4	Charcot-Marie-Tooth disease 4 J	CMT4J	611,228
FKBP5	{Major depressive disorder and accelerated response to antidepressant drugs}	MDD	608,516
FMR1	Fragile X syndrome	FRAX	300,624
FMR1	Fragile X tremor/ataxia syndrome	FXTAS	300,623
GATAD2B	Mental retardation, autosomal dominant 18	MRD18	615,074
HCFC1	Mental retardation, X-linked 3	MRX3	309,541
HNRNPH2	Mental retardation, X-linked, syndromic, Bain type	MRXSB	300,986
HPRT1	Lesch-Nyhan syndrome	LNS	300,322
HSD17B4	Perrault syndrome 1	PRLTS1	233,400
HSPB1	Neuropathy, distal hereditary motor, type IIB	HMN2B	608,634
HSPB1	Charcot-Marie-Tooth disease 2F	CMT2F	606,595
HUWE1	Mental retardation, X-linked 17	MRX17	300,705
HUWE1	Mental retardation, X-linked, syndromic, Turner type	MRXST	300,706
LAS1L	Wilson-Turner syndrome	WTS	309,585
LMNA	Charcot-Marie-Tooth disease 2B1	CMT2B1	605,588
LRPPRC	Leigh syndrome French-Canadian type	LSFC	220,111
MARS	Charcot-Marie-Tooth disease 2 U	CMT2U	616,280
MTHFD1	Neural tube defects, folate-sensitive	NTDFS	601,634
NSUN2	Mental retardation, autosomal recessive 5	MRT5	611,091
PDK3	Charcot-Marie-Tooth disease, X-linked dominant, 6	CMTX6	300,905
PPP2R1A	Mental retardation, autosomal dominant 36	MRD36	616,362
PSMD12	Stankiewicz-Isidor syndrome	STISS	617,516
RANBP2	Encephalopathy, acute, infection-induced, 3	IIAE3	608,033
SAMHD1	Aicardi-Goutieres syndrome 5	AGS5	612,952
SLC25A1	Combined D-2- and L-2-hydroxyglutaric aciduria	D2L2AD	615,182
SLC25A22	Epileptic encephalopathy, early infantile, 3	EIEE3	609,304
SPTLC1	Neuropathy, hereditary sensory and autonomic, type IA	HSN1A	162,400
TECR	Mental retardation, autosomal recessive 14	MRT14	614,020
THOC2	Mental retardation, X-linked 12	MRX12	300,957
TUBB3	Cortical dysplasia, complex, with other brain malformations 1	CDCBM1	614,039
TUBB4A	Leukodystrophy, hypomyelinating, 6	HLD	612,438
USP9X	Mental retardation, X-linked 99	MRX99	300,919
USP9X	Mental retardation, X-linked 99, syndromic, female-restricted	MRXS99F	300,968

### SMCR8 associates with proteins of the ubiquitination and protein decay pathways

Our MS analyses also showed that both SMCR8 and C9orf72 proteins bind the proteasome. Our lists of SMCR8- and C9orf72-interactors were examined for KEGG pathways using the DAVID Functional Annotation Tool (NIAID, NIH, [83]). In the case of SMCR8-associated proteins, proteasome (7.6% of total proteins), RNA transport (6.2%), and protein processing in the endoplasmic reticulum (4.4%) pathways predominated (Fig. S2A). For C9orf72, proteins associated with the proteasome and RNA transport pathways accounted for 4.5 and 8% of the total, respectively, while spliceosome pathway proteins (9%) were most abundant (Fig. S2B). Twenty-six proteasome subunits co-IPed with FL-SMCR8, and 9 subunits were detected within the C9orf72-FL interactome. Furthermore, based on KEGG and Autophagy Database (http://www.tanpaku.org/autophagy/) analyses, 24 proteins involved with phagosome and autophagy pathways were detected in the SMCR8 interactome, including proteins linked with neurological diseases (CDK5, DYNC1H1, HUWE1, PPP2R1A, RANBP2, STUB1, TUBB3, TUBB4A, and USP9X; Tables 1, 2, S2).

Our SMCR8 interactome also contained 9 ubiquitination pathway factors, including ubiquitin ligases and peptidases (Table 1). Therefore, we examined MS-sequenced peptides deriving from immunoprecipitated FL-SMCR8 for ubiquitin modification (72% coverage of the total protein). A total of 9 high confidence modified lysine residues were predicted by at least 5 peptides in two independent experiments, suggesting that SMCR8 is highly ubiquitinated. Eight of these lysines were also identified by at least one of three ubiquitination prediction algorithms, including UbPred [86], BDM-PUB (http://bdmpub.biocuckoo.org), and UbiSite (http://csb.cse.yzu.edu.tw/UbiSite/) (Table S3). We then considered the phylogenic conservation of these lysines by aligning SMCR8 protein sequences from 8 vertebrate (human, chimpanzee, dog, mouse, rat, chicken, zebrafish, and frog) and two mollusc (freshwater snail and sea slug) species (Fig. S3). Eight of the 9 lysines detected by MS as modified were conserved among at least 8 species, including 2 residues (K232, K479) found in both molluscs, suggesting that these post-translational modifications (PTMs) might be functionally relevant.

**Fig. 3:**
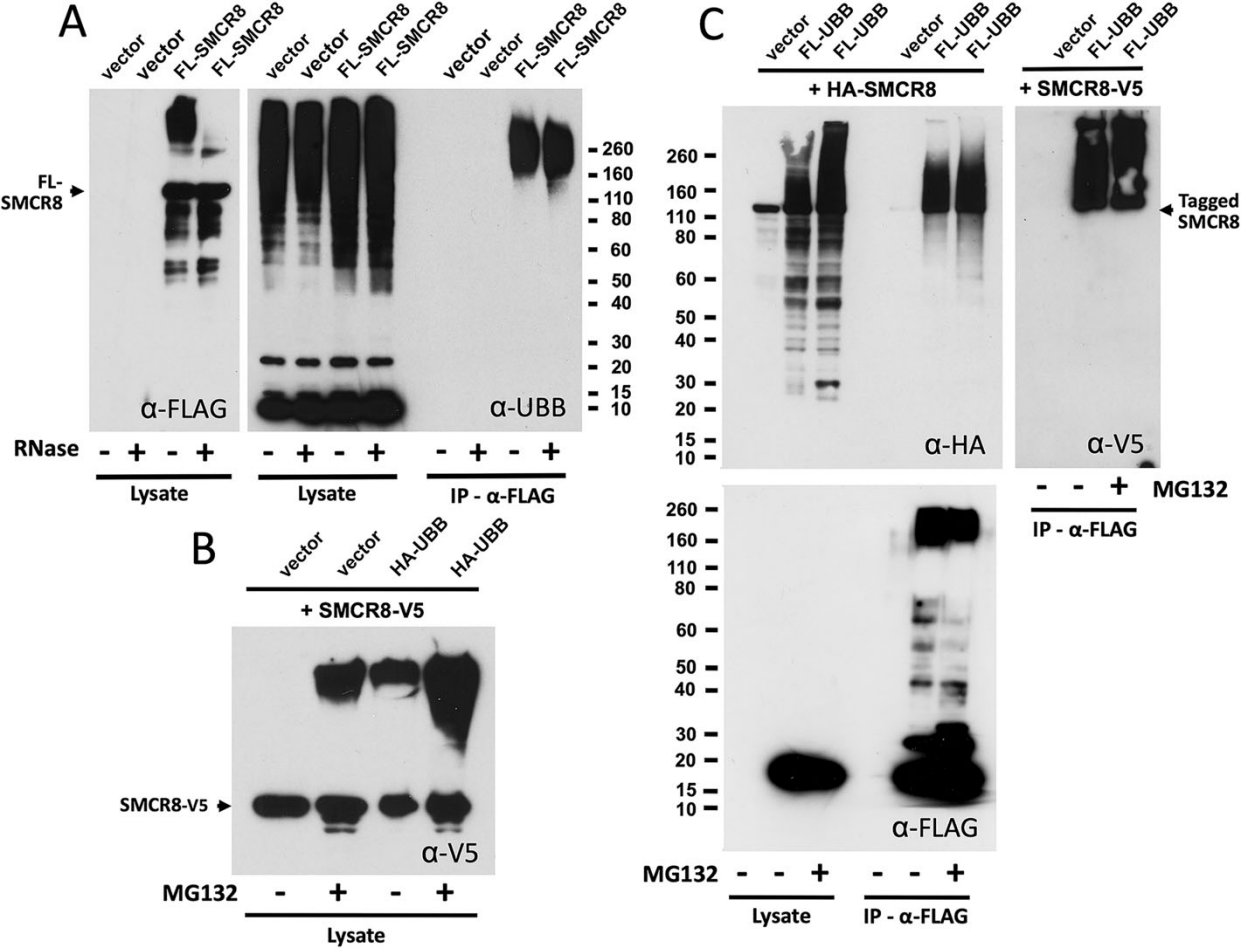
Evidence that SMCR8 protein is poly-ubiquitinated. **a** The FL-SMCR8 construct was transfected in 293T cells and immunoprecipitated with α-FLAG antibody-bound agarose. A Western blot of whole cell lysates probed with α-FLAG antibody shows expression of full-length FL-SMCR8 protein plus HMW products consistent with PTMs (left). Probing with α-UBB antibody marks HMW products in immunoprecipitates consistent with either poly-ubiquitinated FL-SMCR8 protein or the presence of other HMW ubiquitinated proteins that co-IP with the SMCR8 complex (right). IP reactions were in the presence or absence of 50 μg/ml RNases. **b** C-terminal V5-tagged SMCR8 and empty vector or HA-tagged ubiquitin were coexpressed in 293T cells and treated or not treated with the proteasome inhibitor MG132. Expression of SMCR8-V5 protein and empty vector, in the presence but not absence of MG132, produces HMW bands on Western blots that are consistent with post-translational modification of SMCR8 at multiple sites. SMCR8-V5 protein coexpressed with HA-UBB and without MG132 shows the same HMW bands, which increase in signal intensity upon incubation with MG132. **c** V5- or HA-epitope-tagged SMCR8 was coexpressed with empty vector or FLAG-tagged UBB in 293T cells and incubated overnight in the presence or absence of MG132. Cell lysates were subjected to immunoprecipitation with α-FLAG agarose, followed by Western blotting and probing with α-HA (top left panel), α-V5 (top right) or α-FLAG (bottom left) antibodies. A HMW smear seen in immunoprecipitates is consistent with poly-ubiquitination of tagged SMCR8 proteins. In general, overexpression of ubiquitin does not lead to a significant decrease in full-length SMCR8 protein levels

Immunoprecipitating FLAG-tagged SMCR8 and probing with α-ubiquitin on Western blots reveals high-molecular weight (HMW) proteins consistent with polyubiquitinated SMCR8 and/or other large ubiquitinated proteins bound in the SMCR8 complex (Fig. 3a). In whole cell lysates, SMCR8-V5, in the presence of the proteasome inhibitor MG-132 and/or coexpressed ubiquitin, showed HMW products consistent with multiple PTMs (Fig. 3b). Furthermore, FLAG-tagged ubiquitin coimmunoprecipitates on α-FLAG agarose, and so by implication is conjugated to cotransfected HA- or V5-tagged SMCR8 (Fig. 3c). Although treatment with MG132 caused accumulation of HMW SCMR8 protein species, suggesting their regulation by the ubiquitin-proteasome system (UPS), full-length SMCR8 signal was little decreased in the presence of coexpressed ubiquitin (Fig. 3b,c).

**Fig. 4:**
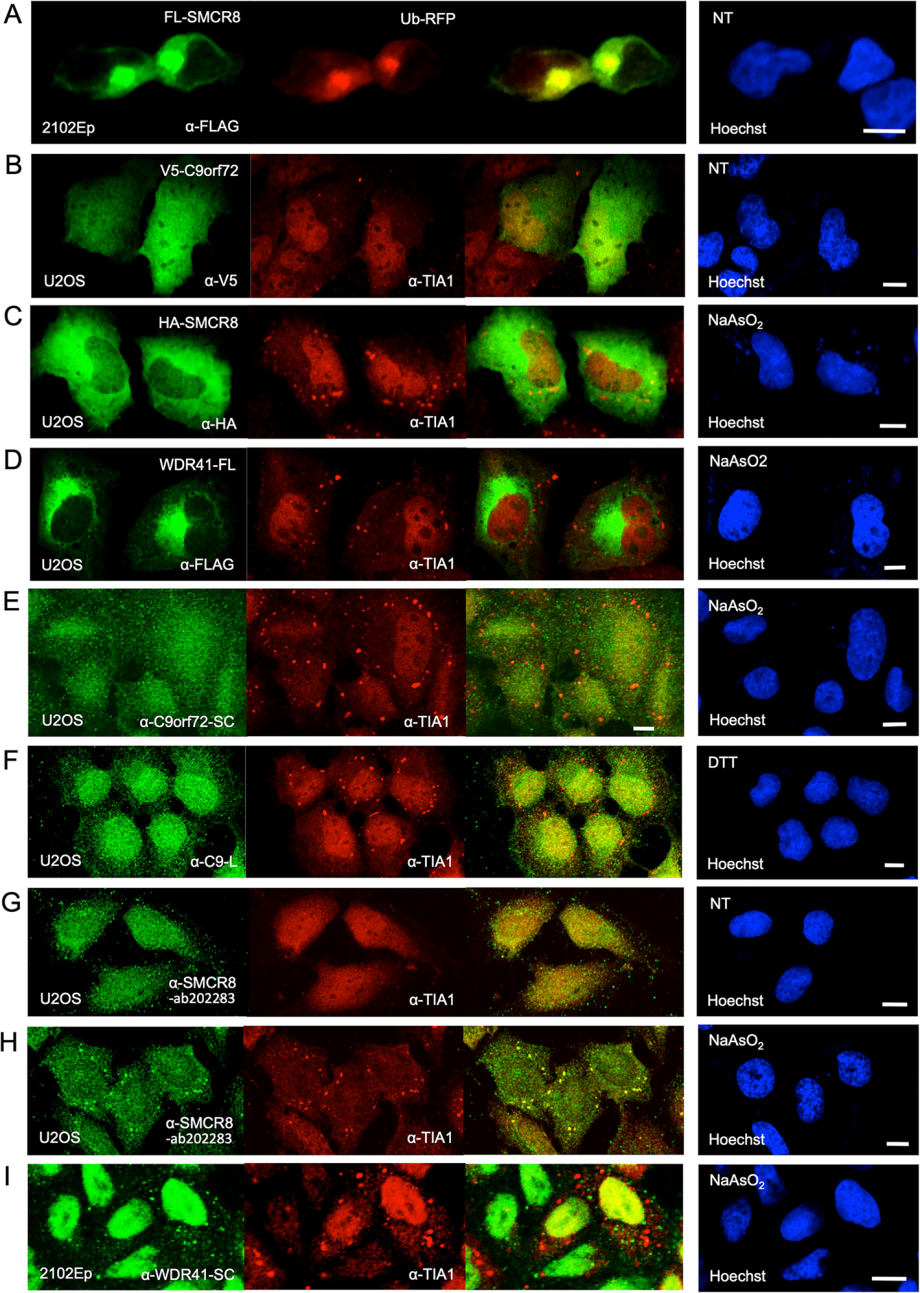
Immunofluorescence microscopy shows evidence for association of endogenous SMCR8 protein with cytoplasmic aggregates. **a** FLAG-tagged SMCR8 and RFP-tagged ubiquitin transfected in 2102Ep cells colocalize in a structure consistent with the aggresome. **b** Overexpression of V5-tagged C9orf72 does not induce stress granule formation in unstressed U2OS cells. **c** Exogenously expressed HA-SMCR8 protein is not observed in SGs of U2OS cells stressed with NaAsO_2_. **d** WDR41-FL protein does not colocalize with SG marker protein TIA1 in U2OS cells stressed with NaAsO_2_. **e** Endogenous C9orf72 protein detercted by the α-SMCR8-SC antibody does not colocalize with SGs in NaAsO_2_-stressed U2OS cells (see also Fig. S4E). **f** Endogenous C9orf72 protein detected by the C9-L antibody [54] does not colocalize with SGs in DTT-stressed U2OS cells. **g**,**h** Endogenous SMCR8 detected by the α-SMCR8-ab202283 antibody localizes to SGs of stressed (**h**), but not unstressed (**g**) U2OS cells (see also Fig. S4G-I). **i** The α-WDR41-SC antibody does not detect endogenous protein in SGs of NaAsO_2_-stressed 2102Ep cells. NT: no treatment. Cell nuclei were stained with Hoechst 33342 (right-most panels). Size bars are 10 μm

Using confocal IF microsopy, we observed that overexpression of red fluorescent protein (RFP)-tagged ubiquitin induces formation of a large aggregate consistent with the aggresome and marked by colocalization with coexpressed and therefore likely UBB-bound FL-SMCR8 (Fig. 4a). Aggresomes appear mainly within an indentation of the nucleus at the microtubule-organizing center and form when the protein-degradation machinery of the cell is overwhelmed [90]. Misfolded and ubiquitinated proteins, including perhaps SMCR8, are transported to the aggresome along the microtubule network by means of the dynein motor complex (which includes cytoplasmic dyneins DYNC1H1 and DYNC1I2, both detected in FL-SMCR8 iimmunoprecipitates, Table S2). An alternative ubiquitin-independent pathway involves interaction of STUB1 and BAG3, which transfer misfolded proteins to heat shock protein 70 (all proteins that co-IPed with FL-SMCR8, Table S2) and the dynein motor complex to promote formation of aggresomes [91, 92].

Thus, SMCR8 protein is bound by ubiquitin and may recruit UPS complexes to the vicinity of its other associated cellular proteins, numbers of which have been linked with neuropathologies (Table 2).

### Evidence that endogenous SMCR8 accumulates in cytoplasmic stress granules

The accumulation of neuronal RNA and protein aggregates, including cytoplasmic stress granules, is a pathogenic hallmark of a number of neurodegenerative diseases, among them FTD and ALS [93–95]. SGs assemble rapidly under cellular stress and include the small, but not large, ribosomal subunits bound to translation initiation factors such as eIF2 and eIF3 (reviewed in [96]). Processing-bodies (PBs) and SGs are dynamic cytoplasmic aggregates that participate in mRNA decay, and SGs in mammalian cells are heavily ubiquitinated [97]. Because previous publications implicated C9orf72 protein expression in the metabolism of SGs [88, 98], we wished to determine if the C9orf72 binding partner SMCR8 associates with SGs in various tumor cell lines.

As reported by others [28], we observed epitope-tagged SMCR8 and C9orf72 proteins to both have a diffuse cytoplasmic distribution with protein also observed in nuclei, although nuclear localization was more evident for C9orf72 (Fig. 4b,c, S4A-C). However, although Maharjan et al. [98] reported that SGs were induced in a majority of unstressed mouse Neuro2A (N2A) neuroblastoma cells when transfected with myc-tagged C9-L, we failed to observe this phenomenon for tagged C9-L or SMCR8 proteins transfected alone or in combination (not shown) in unstressed human osterosarcoma U2OS, HEK 293T, or neuroblastoma cell lines (Fig. 4b, S4B). Furthermore, when cells were treated with 250 μM of the oxidative stressor sodium arsenite (NaAsO_2_) for 80 min, tagged C9orf72, SMCR8, or WDR41 protein very rarely colocalized in aggregates with endogenous canonical SG marker protein TIA1 in multiple cell lines (Figs. 4c,d, S4C).

We next examined localization of endogenous C9orf72 and SMCR8 proteins in cells. The α-C9orf72-SC and α-C9orf72-PT antibodies both detected nuclear and cytoplasmic distribution for C9orf72 protein, with fine cytoplasmic granululazion visible in unstressed cells that was more evident for the latter antibody (Fig. S4D). However, contrary to previous studies that used these antibodies to report SG localization [88, 98], we failed to detect endogenous C9orf72 in stress-induced U2OS (Figs. 4e, S4E) or 2102Ep (not shown) cells, although C9orf72 infrequently justaposed or overlapped with SGs and/or PBs in N2A cells (Fig. S4F.G). To confirm further these observations, two polyclonal antibodies developed by the Robertson lab [19, 79], and specific for C9-L (Fig. 4f) and C9-S (not shown) isoforms, were also tested but failed to show obvious C9orf72 protein presence in TIA1-marked SGs in DTT- or NaAsO_2_-stressed cells of multiple lines, including U2OS and 2012Ep cells. Thus, detection of C9orf72 in SGs appears to be cell line and possibly antibody dependent.

We also used the α-SMCR8-PT and α-SMCR8-ab202283 antibodies to examine endogenous SMCR8 protein localization. In unstressed U2OS cells, endogenous SMCR8 was nuclear and more prominently cytoplasmic with speckled staining (Fig. 4g). However, when cells were stressed with NaAsO_2_, SMCR8 redistributed to large intensely staining foci that colocalized with TIA1 (Fig. 4h). Fig. S4H shows SMCR8 protein in large cytoplasmic aggregates of NaAsO_2_-stressed HEK 293T cells that costain with a different endogenous SG marker, the LINE-1 retrotransposon-encoded ORF1 protein [81], while Fig. S4I shows costaining with SG-marker eIF3η in human neuroblastoma SK-N-SH cells. In N2A cells treated with the endoplasmic reticulum stressor thapsigargin, SMCR8 granules were marked by a p70 S6 kinase antibody known to recognize HEDLS/EDC4, a PB component (Fig. S4J, [77]): PBs frequently overlap or juxtapose with SGs in stressed cells [99]. Endogenous SMCR8 granules in unstressed N2A cells also partially colocalized with GW182 autoantigen, which marks PBs (Fig. S4K) [80]. However, as noted above, SMCR8 commercial antibodies detect multiple protein species (Fig. S1B-E), some possibly non-specific, and we cannot be certain that canonical full-length endogenous SMCR8 proteins are what we see in SGs. Nevertheless, our data suggest that in stressed cells a fraction of endogenous SMCR8 protein is directed to cytoplasmic SGs.

Our analyses showed that TAR DNA binding protein 43 (TDP-43, product of the TARDBP gene) binds SMCR8 (Fig. 2; Table 2). Mutations in TARDBP are involved in about 4% of familial and 1% of sporadic ALS (sALS) cases. However, even wild-type TDP-43, while mostly nuclear in healthy cells, is cleaved and hyperphosphorylated and accumulates in ubiquitinated cytoplasmic aggregates in neurons of almost all ALS and about half of FTLD patients (reviewed in [100]). We tested if endogenous or overexpressed SMCR8 protein colocalizes with TDP-43 protein in cytoplasmic granules but found this not to be the case in unstressed or stressed U2OS or 2102Ep cells (Fig. S4L).

Hexanucleotide expansions within transcripts of the C9orf72 ALS gene may undergo non-conventional repeat-associated non-ATG (RAN) translation and generate dipeptide repeats that aggregate in the cytoplasm of neuronal cells of C9ALS patients (reviewed in [101]). To see if such aggregates might colocalize with SMCR8, we coexpressed in 293T cells FL-SMCR8 and a C9orf72 RAN translation product of 50 GA-dipeptide repeats tagged with EGFP [68]. Overexpressed dipeptide proteins formed one to three large cytoplasmic aggrgates in each cell that were were ringed by, but mostly excluded SMCR8 (Fig. S4M).

Finally, the α-WDR41-SC antibody marks WDR41 protein as predominantly nuclear but also with faint cytoplasmic granules that fail to colocalize with SGs in unstressed or stressed U2OS, 2102Ep, 293T, or N2A cells (Fig. 4i and not shown). On the other hand, the α-WDR41-PT antibody colocalizes with a minor subset of granules positive for 4-ET, a marker of PBs (Fig. S4N). However, while the α-WDR41-SC antibody recognizes only bands consistent in size with WDR41 isoforms in HEK 293T, 2102Ep, and SK-N-SH cells (Fig. S1F), the α-WDR41-PT antibody detects other non-canonical protein species (Fig. S1G), and the specificity of its SG staining is thus uncertain.

Searching the Mammalian Stress Granules Proteome Database (https://msgp.pt) [102], we found that 18% of the SMCR8 proiein interactome (61/340) and 26% (35/201) of the C9orf72 interactome are known SG-associated proteins. It is thus possible that SG components bind endogenous SMCR8-C9orf72 complexes and shepherd them to SGs, although why this would not also be the case for overexpressed exogenous SMCR8 or C9orf72 proteins is unclear.

### SMCR8 expression in ALS patient brain tissues

**Fig. 5:**
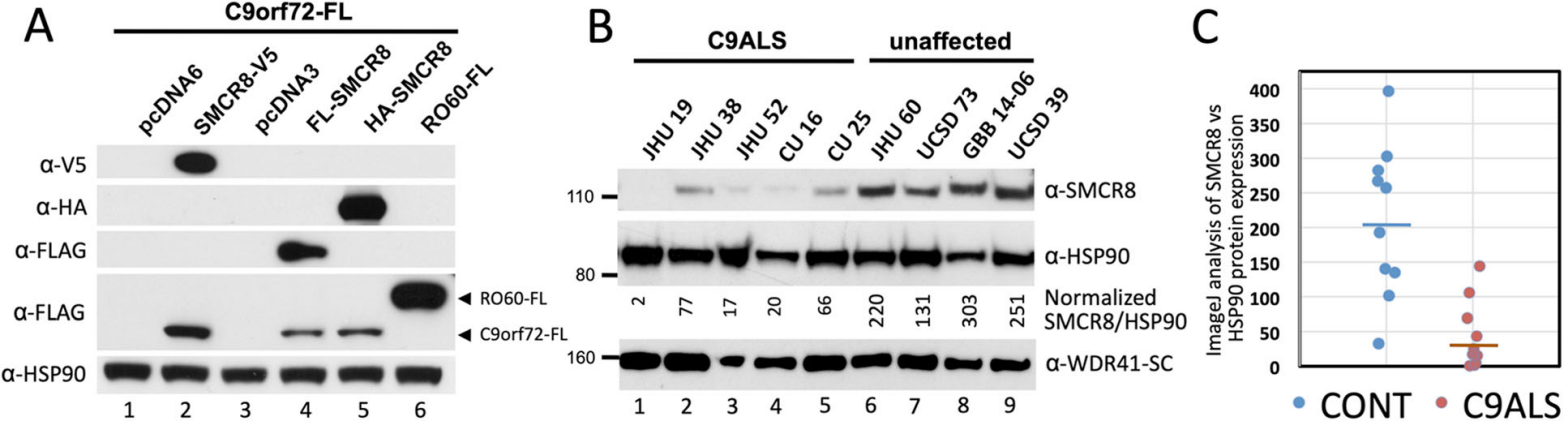
Expression of C9orf72 and SMCR8 proteins are positively correlated in cell lines and human brain tissues. **a** C9orf72-FL was coexpressed in HEK 293T cells with 3 different epitope-tagged SMCR8 constructs, FLAG-tagged RO60 protein, or empty vectors (pcDNA3 and pcDNA6 myc/his B). A Western blot of whole cell lysates was probed sequentially with rb α-FLAG, ms α-HA, ms α-V5, and rb α-HSP90 antibodies, the latter as a loading control. At the exposure time for the film shown, expression of C9orf72-FL was not seen in the presence of empty vector or RO60-FL, but signal was robust in the presence of SMCR8. **b** Western blot of brain motor cortex tissue lysates of C9ALS patients (lanes 1–5) and unaffected control individuals (lanes 6–9) probed with α-SMCR8 and α-HSP90 antibodies. Sample names are shown above the panels (see Table S4). Numbers below the middle panel are normalized ratios of SMCR8 to HSP90 expression determined by ImageJ analysis of band intensities and calculated as described in the text. The lower panel shows the approximately 150-kD unspecified band detected by α-WDR41-SC antibody in human brain tissue lysates (see Fig. S1F): this panel is included only as an additional loading control and is not intended to show expression of canonical WDR41 protein. Approximtely 50 μg of protein was loaded in each lane. **c** Dot plot of ratios of SMCR8 to HSP90 protein band intensities determined by ImageJ analyses of brain tissues lysates from 11 C9ALS and 10 control individuals. Each sample point is the average of 2 to 4 independent Western blot analyses. A short horizontal line indicates mean values. The presence of a C9orf72 hexanucleotide expansion in each C9ALS carrier individual was confirmed by Columbia University and Target ALS using RP-PCR and Illumina Expansion Hunter, but expansion copy numbers are not known

Despite its strong association with protein-degradation factors, SMCR8 overexpression does not stimulate degradation of C9orf72 protein with which it is in complex. Contrarily, multiple studies in cells and knockout mice have shown that protein but not RNA levels of *SMCR8* and *C9orf72* are positively correlated, suggesting that in complex the two proteins stabilize and protect each other from degradation [26, 28, 29, 32, 47, 54, 65, 103]. On the other hand, increased SMCR8 protein reportedly has little effect on WDR41 levels in KO mice or cells [32, 35]. We confirmed in 293T cells that overexpression of SMCR8 with various tags strongly increased levels of cotransfected FL-C9-L protein, while cotransfection of empty vectors or an unrelated protein (RO60) did not (Fig. 5a). Considering the interplay between SMCR8 and C9orf72 proteins, and the fact that *C9orf72* RNA expression is reduced in some C9ALS patient cohorts, we asked if SMCR8 expression levels are altered in the brains of C9ALS patients compared with non-affected controls.

We first examined transcription levels of *C9orf72*, *SMCR8*, and *WDR41* genes in RNA-Seq datasets from several sequence read archives that contain C9ALS sample data. GEO dataset GSE67196 includes cerebellum and frontal cortex samples of 9 healthy, 8 C9ALS, and 10 sALS individuals. Using TEtranscripts [84] to analyze *C9orf72* gene expression levels, we found a significant log_2_ 0.96-fold decrease (padj 4.6E-5) in the frontal cortex of C9ALS vs sALS individuals and a 1.1-fold decrease (padj 1.6-E4) in the cerebellum of C9ALS vs control individuals; however, in neither case was decrease in *SMCR8* expression significant. The NeuroLINCS dbGaP Study phs001231 (SRP098831) consists of poly(A) + non-stranded mRNA of iPSC-derived motor neurons from 4 C9ALS, 3 spinal muscular atrophy (SMA), and 3 unaffected individuals (2 or 3 replicates each). No significant changes in *C9orf72* or *SMCR8* transcript levels were seen in this dataset, although *WDR41* sequence read numbers were reduced about 0.35-fold in both C9ALS vs control and SMA vs control samples (padj< 0.01). Finally, a recent RNA-Seq study comparing C9 FTLD and FTLD/motor neuron disease patients with unaffected control individuals reported a highly significant decrease in *C9orf72* RNA levels in C9 FTLD samples; however, this data showed no significant change in *SMCR8* or *WDR41* RNA expression [24].

We next assayed endogenous SMCR8 protein expression levels in the context of the *C9orf72* hexanucleotide expansion. Motor cortex brain tissue lysate samples of 11 C9ALS and 10 unaffected control individuals were analyzed by Western blotting with α-SMCR8 antibodies (Fig. 5b, Table S4). Multiple film exposures were made to optimize signal to noise. Individual band intensities were quantitated with ImageJ software [104] and normalized against the summed exposures of all equivalent bands on the same gel. SMCR8 signal was then normalized to endogenous HSP90 protein signal detected on the same gel after reprobing with α-HSP90 antibody. Remarkably, an average 5-fold reduction in SMCR8 protein signal was seen in C9ALS vs control tissues (Fig. 5b). We also tested by Western blotting cerebrospinal fluid samples from 5 C9ALS patients and 5 unaffected controls, but were unable to detect full-length SMCR8 protein signal with either the α-SMCR8-PT or α-SMCR8-ab202283 antibodies (not shown). We also plotted normalized SMCR8 protein signal against ALS disease duration in months (Table S4), finding a weak negative but non-significant correlation (*r* = 0.34). Nevertheless, altogether our data recommend further investigation of SMCR8 protein level as a potential biomarker of the *C9orf72* expansion disease mutation.

## Discussion

In this study we characterized the SMCR8 protein interactome and found it to include numerous components of the ubiquitin-proteasome system, including ubiquitin ligases and peptidases. Of note, the IP method used here exploited FLAG-tagged proteins and so overcame limitations imposed by differences in isoform expression and non-specific protein species recognized by C9orf72 and SMCR8 antibodies. Despite evidence that SMCR8 itself is ubiquitinated at multiple residues, its degradation is not significantly induced in the presence of overexpressed ubiquitin suggesting other roles linking it with the UPS. Recruitment of UPS components to autophagy complexes could be one such role, and our SMCR8 interactome contains 24 autophagy pathway-associated proteins (Table 1). Ubiquitin plays a fundamental role not only in proteasome-mediated protein degradation but also in the targeting of proteins for degradation by autophagic complexes. Protein ubiquitination also regulates multiple steps of the autophagy pathway (reviewed in [105, 106]). For example, E3 ubiquitin ligase STUB1, a protein that co-IPs with SMCR8 (Table 1, Fig. 2), regulates autophagy by targeting TFEB for degradation by the UPS [107]. Also, E3 ligase HUWE1 (Table 1) mediates the ubiquitination and proteasomal degradation of WIPI2, a protein involved in autophagosome formation [108].

Recruitment of ubiquitination factors would be consistent with reported roles of the C9orf72-SMCR8 complex in autophagy and endosomal-lysosomal metabolism. The C9orf72/SMCR8/WDR41 complex associates with the ATG8 autophagy receptor network and influences activity of the ULK1-RB1CC1-ATG13-ATG101 autophagy initiation complex (RB1CC1 was detected in both our SMCR8 and C9orf72 protein interactomes, Tables S1, S2) [22, 28, 29, 31, 34]. C9orf72 and SMCR8 reportedly also play roles in regulating mTORC1 and TFEB autophagy and lysosomal gene transcription factors upstream of autophagy [26, 31, 32, 34, 57, 103, 109, 110]. Ugolino et al. [32], however, indicated a negative effect of C9orf72 on autophagy, and Yang et al. [31] reported increased autophagic flux in C9orf72 knockdown MEF cells, opposite to the reduction they saw in SMCR8-deficient cells. Unlike some *C9orf72*-deficient mice [45], *Smcr8* KO mice showed motor behavior defects, including axonal swelling due to impaired autophagy and motor neuron axonal transport of autophagosomes [25]. Apparently, although in complex, some biological roles of C9orf72 and SMCR8 proteins differ.

Association of the SMCR8-C9orf72 complex with the UPS and autophagy would also be consistent with stress granule localization, since protein ubiquitination regulates SG dynamics. Components of the UPS, including ubiquitin, co-localize with SGs, while proteasome inhibition, and consequent increase in ubiquitinated proteins, induces SG formation [111–113]. Recent evidence also suggests SGs are regulated by autophagy [114, 115], and it has been proposed that improper metabolism of SGs could be involved in ALS pathology [93, 94]. Interestingly, Chitiproulu et al. [88] proposed that C9orf72 protein associates with autophagy cargo receptor p62 (encoded by the *SQSTM1* gene) to control SG elimination rather than assembly by forming a complex that eliminates by autophagy SG proteins dimethylated on arginines (of note, we found p62 in the SMCR8 but not C9orf72 interactomes; Fig. 2, Table 2S).

However, our data disagree in some aspects with previously published results concerning C9orf72 colocalization with SGs. While, Maharjan et al. [98] reported that overexpression of myc-tagged C9-L led to the spontaneous appearance of SGs in a majority of N2A cells and cortical neurons in the absence of cellular stress, we failed to reproduce these observations in either U2OS or N2A cells for tagged C9orf72 or SMCR8 proteins, overexpressed together or separately. Furthermore, using the α-C9orf72-PT antibody (Fig. S1A), Maharjan et al. [98] noted that endogenous C9orf72 protein colocalized with a fraction of SGs in neuronal cell lines and cortical neurons in response to DTT and heat shock-induced cell stress, and that C9orf72 depletion inhibited SG assembly, impaired expression of proteins required for their formation, and increased cell sensitivity to stress. However, despite testing several antibodies, cell lines and conditions, we could not detect endogenous C9orf72 in SGs of selected non-neuronal cancer-derived cell lines, and we saw only minor colocalization of C9orf72 with SGs and PBs of N2A cells. Thus, association of C9orf72 protein to SGs appears to be cell line-dependent.

On the other hand, we observed endogenous, but not exogenously expressed, SMCR8 protein localization to SGs of all chemically stressed cell lines tested. Interestingly, about one-fifth of the putative interacting proteins we identified as members of our C9orf72 and SMCR8 interactomes are known SG proteins, which themselves might play a role in targeting of SMCR8 complexes to granules. It is conceivable that SMCR8-C9orf72 SG association is sensitive to cell type, cellular conditions, and levels of interacting proteins as determinants of entry into SGs, and perhaps these factors explain discrepancies between our data and previously published observations.

As reported in other studies, we also presented supporting evidence that C9orf72 protein levels are positively correlated with those of SMCR8 in cultured cells [26, 28, 29, 32, 47, 54, 65, 103]. Furthermore, we now show that SMCR8 protein expression is reduced in the brains of C9ALS patients compared with unaffected controls (and as also recently noted by [25]. To date, it has been reported that a small number of proteins, including neurofilament proteins, are differentially expressed in the CSF of ALS and FTD proteins and have been proposed as candidate biomarkers for the C9orf72 mutation [116, 117]. Whether or not SMCR8 protein can also be an effective CSF or plasma biomarker for C9 expansion patients remains to be determined and is likely contingent upon the development of better α-SMCR8 antibodies.

## Conclusions

In this study we characterized the protein interactome of SMCR8, which binds the protein product of *C9orf72*, the major susceptibility gene for ALS. Using a robust and highly specific protocol, we demonstrated ubiquitination without significant degradation of SMCR8 protein and its association with many components of the ubiquitin-proteasome system. Evidence was presented for localization of endogenous SMCR8 protein to cytoplasmic stress granules, although in several cell lines we failed to reproduce previous observations that C9orf72 protein enters these granules. SMCR8 protein levels were downregulated in whole tissue brain lysates of C9ALS patients compared with unaffected controls, suggesting the potential usefulness for SMCR8 as a biomarker of the disease state.

In addition to ALS and FTD, the *C9orf72* gene expansion mutation has been linked with other neurodegenerative and psychiatric disorders, although etiological roles remain unknown [118–123]. We have shown that SMCR8, whose cellular levels positively correlate with C9orf72 protein expression, associates not only with many factors of protein metabolism and stress granule dynamics, but also with numerous products of genes linked with a range CNS disorders (65/340 in total, Table 2). It is therefore reasonable in future studies to consider a role for SMCR8 in these diverse neuropathologies, perhaps relating to recruitment of the UPS with consequent effects on protein homeostasis.

## Supplementary information

**Additional file 1 Fig. S1.** Western blotting of cell line and brain tissue lysates using commercial antibodies against C9orf72, SMCR8, and WDR41 proteins. Antibodies shown are (A) α-C9ORF72-PT (Proteintech Group 22637–1), (B) α-SMCR8-PT (Proteintech 21125–1-AP, (C) α-SMCR8-B (Bethyl Laboratories A304-694A), (D) α-SMCR8-ab186504 (Abcam ab186504), (E) α-SMCR8-ab202283 (Abcam ab202283), (F) α-WDR41-SC (Santa Cruz sc-137923), and (G) α-WDR41-PT (Proteintech 26817–1-AP). Large arrows mark full-length protein products. The small arrows in (F) and (G) mark the presumed minor 45.5 kD WDR41 isoform. Samples were run on NuPAGE 4–12% Bis-Tris Protein gels (Novex) and Western blotting was performed according to [81, 82]. Molecular weight markers are Novex Sharp Pre-stained Protein Standard. Motor: motor cortex, Occ: occipital cortex, S.C.: spinal cord; sample identifier numbers are also shown. **Fig. S2.** Pie chart of results of DAVID (Database for Annotation, Visualization and Integrated Discovery, [83]) analyses of KEGG pathways showing selected functional categories for candidate member proteins of the (A) FL-SMCR8 and (B) C9orf72-FL protein interactomes. Percentages of the total number of proteins identified (Tables S1 and S2) for each category are shown within the slices. **Fig. S3.** Phylogenetic multi-sequence alignment of SMCR8 protein sequences for ten species. Alignments were made with Clustal Omega 1.2.1 (EMBL-EBI) followed by BoxShade 3.2 (http://sourceforge.net/projects/boxshade). Pink shading marks amino acid residues identical in at least 8 species, while green includes conservative replacements. Lysine residues predicted by MS sequencing to be ubiquitinated are boxed in blue (see Table S3). Species shown are: *Homo sapiens*, human; *Pan troglodytes*, chimpanzee; *Canus lupus familiaris*, dog; *Mus musculus*, house mouse; *Rattus norvegicus*, brown rat; *Gallus gallus domesticus*, chicken; *Danio rerio*, zebrafish; *Xenopus tropicalis*, western clawed frog; *Biomphalaria glabrata*, freshwater snail; *Aplysia californica*, California sea hare. **Fig. S4.** Immunofluorescence microscopy evidence for association of SMCR8 protein with cytoplasmic aggregates. (A) V5-tagged SMCR8 does not significantly enter SGs of NaAsO_2_-stressed N2A cells. (B) Overexpression of FLAG-tagged C9orf72 (left) or SMCR8 (right) protein does not induce formation of cytoplasmic granules in neuroblastoma cells. (C) V5-tagged exogenously expressed C9orf72 fails to enter SGs in NaAsO_2_-treated U2OS cells. (D) Endogenous C9orf72 protein speckles observed in unstressed N2A cells are not marked by TIA1, a SG protein. (E) C9orf72 protein detected by the α-C9orf72-PT antibody fails to enter SGs of NaAsO_2_-stressed U2OS cells. (F,G), Endogenous C9orf72 protein detected by the α-C9orf72-SC antibody colocalizes or justaposes with only a minor subset of SGs (marked by TIA1) or PBs (marked by 4-ET) in stressed mouse neuroblastoma N2A cells (see arrows). (H) In NaAsO_2_-stressed 293T cells, endogenous SMCR8 detected by the α-SMCR8-PT antibody colocalizes with granules marked by ORF1p, a protein encoded by LINE-1 retrotransposons [81]. (I) In NaAsO_2_-stressed human neuroblastoma SK-N-SH cells, endogenous SMCR8 localizes to SGs marked by α-eIF3η antibody. (J) In N2A cells treated with the endoplasmic reticulum stressor thapsigarin, endogenous SMCR8 protein colocalizes to granules marked by HEDLS/EDC4 (detected by α-p70 S6 kinase antibody, [77]), a component of PBs. (K) In unstressed N2A cells, endogenous SMCR8 localizes in granules with GW142, a PB marker. (L) Exogenously expressed TDP-43 and endogenous SMCR8 proteins do not colocalize in cytoplasmic granules of stressed U2OS cells. (M) SMCR8 rings but is generally excluded from overexpressed GFP-(GA)_50_ dipeptide aggregates. (M) In 293T cells, the α-WDR41-PT antibody detects endogenous protein in or beside a subset granules marked by 4-ET (see arrows). NT: no treatment. Cell nuclei were stained with Hoechst 33342 (right-most panels). Size bars are 10 μm. **Table S1.** The C9orf72 protein interactome determined by mass spectrometry. **Table S2.** The SMCR8 protein interactome determined by mass spectrometry. **Table S3.** Ubiquitinated SMCR8 lysine residues determined by MS sequencing, prediction algorithms, and the comparative phylogenetic analyses of **Fig. S3.Table S4.** Post-mortem brain motor cortex tissue samples used for the analyses of Fig. 5b,c.

## Data Availability

Raw MS sequencing data used to generate Supplementary Tables S1 and S2 will be made available from the corresponding author upon request.

## Abbreviations

ALS: Amyotrophic lateral sclerosis
C9ALS: C9orf72 repeat expansion ALS
CNS: Central nervous system
CSF: Cerebrospinal fluid
FTD: Frontotemporal dementia
FTLD: Frontotemporal lobar degeneration
GEF: Guanine nucleotide exchange factor
GFP: Green fluorescent protein
HMW: High molecular weight
IF: Immunofluorescence
IP: Immunoprecipitation
iPSC: Induced pluripotent stem cell
kD: Kilodalton
N2A: Neuro2A
ORF: Open reading frame
PB: Processing (P-) body
PCR: Polymerase chain reaction
PTM: Post-translational modification
RFP: Red fluorescent protein
sALS: Sporadic ALS
SG: Stress granule
UPS: Ubiquitin-proteasome system
