# Supplementary material for: C9orf72-associated SMCR8 protein binds in the ubiquitin pathway and with proteins linked with neurological disease
Source: Acta Neuropathol Commun. 2020 Jul 16;8:110. doi: 10.1186/s40478-020-00982-x (PMC7364817; doi:10.1186/s40478-020-00982-x)
Supplement: Supplementary file 1 — Additional file 1 Fig. S1. Western blotting of cell line and brain tissue lysates using commercial antibodies against C9orf72, SMCR8, and WDR41 proteins. Antibodies shown are (A) α-C9ORF72-PT (Proteintech Group 22637–1), (B) α-SMCR8-PT (Proteintech 21125–1-AP, (C) α-SMCR8-B (Bethyl Laboratories A304-694A), (D) α-SMCR8-ab186504 (Abcam ab186504), (E) α-SMCR8-ab202283 (Abcam ab202283), (F) α-WDR41-SC (Santa Cruz sc-137923), and (G) α-WDR41-PT (Proteintech 26817–1-AP). Large arrows mark full-length protein products. The small arrows in (F) and (G) mark the presumed minor 45.5 kD WDR41 isoform. Samples were run on NuPAGE 4–12% Bis-Tris Protein gels (Novex) and Western blotting was performed according to [81, 82]. Molecular weight markers are Novex Sharp Pre-stained Protein Standard. Motor: motor cortex, Occ: occipital cortex, S.C.: spinal cord; sample identifier numbers are also shown. Fig. S2. Pie chart of results of DAVID (Database for Annotation, Visualization and Integrated Discovery, [83]) analyses of KEGG pathways showing selected functional categories for candidate member proteins of the (A) FL-SMCR8 and (B) C9orf72-FL protein interactomes. Percentages of the total number of proteins identified (Tables S1 and S2) for each category are shown within the slices. Fig. S3. Phylogenetic multi-sequence alignment of SMCR8 protein sequences for ten species. Alignments were made with Clustal Omega 1.2.1 (EMBL-EBI) followed by BoxShade 3.2 (http://sourceforge.net/projects/boxshade). Pink shading marks amino acid residues identical in at least 8 species, while green includes conservative replacements. Lysine residues predicted by MS sequencing to be ubiquitinated are boxed in blue (see Table S3). Species shown are: Homo sapiens, human; Pan troglodytes, chimpanzee; Canus lupus familiaris, dog; Mus musculus, house mouse; Rattus norvegicus, brown rat; Gallus gallus domesticus, chicken; Danio rerio, zebrafish; Xenopus tropicalis, western clawed frog; Biomphalaria glabrata, freshw [file 40478_2020_982_MOESM1_ESM.pdf]

C9orf72-associated SMCR8 protein binds in the ubiquitin pathway and with proteins linked with neurological disease.

Supplemental Figure Legends

Fig. S1. Western blotting of cell line and brain tissue lysates using commercial antibodies against C9orf72, SMCR8, and WDR41 proteins. Antibodies shown are (A) α-C9ORF72-PT (Proteintech Group 22637-1), (B) α-SMCR8-PT (Proteintech 21125-1-AP, (C) α-SMCR8-B (Bethyl Laboratories A304-694A), (D) α-SMCR8-ab186504 (Abcam ab186504), (E) α-SMCR8-ab202283 (Abcam ab202283), (F) α-WDR41-SC (Santa Cruz sc-137923), and (G) α-WDR41-PT (ProteinTech 26817-1-AP). Large arrows mark full-length protein products. The small arrows in (F) and (G) mark the presumed minor 45.5 kD WDR41 isoform. Samples were run on NuPAGE 4-12% Bis-Tris Protein gels (Novex) and Western blotting was performed according to [81, 82]. Molecular weight markers are Novex Sharp Pre-stained Protein Standard. Motor: motor cortex, Occ: occipital cortex, S.C.: spinal cord; sample identifier numbers are also shown.

Fig. S2. Pie chart of results of DAVID (Database for Annotation, Visualization and Integrated Discovery, [83]) analyses of KEGG pathways showing selected functional categories for candidate member proteins of the (A) FL-SMCR8 and (B) C9orf72-FL protein interactomes. Percentages of the total number of proteins identified (Tables S1 and S2) for each category are shown within the slices.

Fig. S3. Phylogenetic multi-sequence alignment of SMCR8 protein sequences for ten species. Alignments were made with Clustal Omega 1.2.1 (EMBL-EBI) followed by BoxShade 3.2 (http://sourceforge.net/projects/boxshade). Pink shading marks amino acid residues identical in at least 8 species, while green includes conservative replacements. Lysine residues predicted by MS sequencing to be ubiquitinated are boxed in blue (see Table S3). Species shown are: *Homo sapiens*, human; *Pan troglodytes*, chimpanzee; *Canus lupus familiaris*, dog; *Mus musculus*, house mouse; *Rattus norvegicus*, brown rat; *Gallus gallus domesticus*, chicken; *Danio rerio*, zebrafish; *Xenopus tropicalis*, western clawed frog; *Biomphalaria glabrata*, freshwater snail; *Aplysia californica*, California

sea hare.

Fig. S4. Immunofluorescence microscopy evidence for association of SMCR8 protein with cytoplasmic aggregates. (A) V5-tagged SMCR8 does not significantly enter SGs of $NaAsO_2$-stressed N2A cells. (B) Overexpression of FLAG-tagged C9orf72 (left) or SMCR8 (right) protein does not induce formation of cytoplasmic granules in neuroblastoma cells. (C) V5-tagged exogenously expressed C9orf72 fails to enter SGs in $NaAsO_2$-treated U2OS cells. (D) Endogenous C9orf72 protein speckles observed in unstressed N2A cells are not marked by TIA1, a SG protein. (E) C9orf72 protein detected by the α-C9orf72-PT antibody fails to enter SGs of $NaAsO_2$-stressed U2OS cells. (F,G), Endogenous C9orf72 protein detected by the α-C9orf72-SC antibody colocalizes or justaposes with only a minor subset of SGs (marked by TIA1) or PBs (marked by 4-ET) in stressed mouse neuroblastoma N2A cells (see arrows). (H) In $NaAsO_2$-stressed 293T cells, endogenous SMCR8 detected by the α-SMCR8-PT antibody colocalizes with granules marked by ORF1p, a protein encoded by LINE-1 retrotransposons [81]. (I) In $NaAsO_2$-stressed human neuroblastoma SK-N-SH cells, endogenous SMCR8 localizes to SGs marked by α-eIF3η antibody. (J) In N2A cells treated with the endoplasmic reticulum stressor thapsigarin, endogenous SMCR8 protein colocalizes to granules marked by HEDLS/EDC4 (detected by α-p70 S6 kinase antibody, [77]), a component of PBs. (K) In unstressed N2A cells, endogenous SMCR8 localizes in granules with GW142, a PB marker. (L) Exogenously expressed TDP-43 and endogenous SMCR8 proteins do not colocalize in cytoplasmic granules of stressed U2OS cells. (M) SMCR8 rings but is generally excluded from overexpressed GFP-$(GA)_{50}$ dipeptide aggregates. (M) In 293T cells, the α-WDR41-PT antibody detects endogenous protein in or beside a subset granules marked by 4-ET (see arrows). NT: no treatment. Cell nuclei were stained with Hoechst 33342 (right-most panels). Size bars are 10 µm.

Supplemental Tables

Table S1. The C9orf72 protein interactome determined by mass spectrometry.

Table S2. The SMCR8 protein interactome determined by mass spectrometry.

Table S3. Putative ubiquitinated SMCR8 lysine residues determined by MS sequencing, ubiquitination prediction programs, and the comparative phylogenetic analyses of Fig. S3.

Table S4. Post-mortem brain motor cortex tissue samples used for the analyses of Fig. 5b,c.

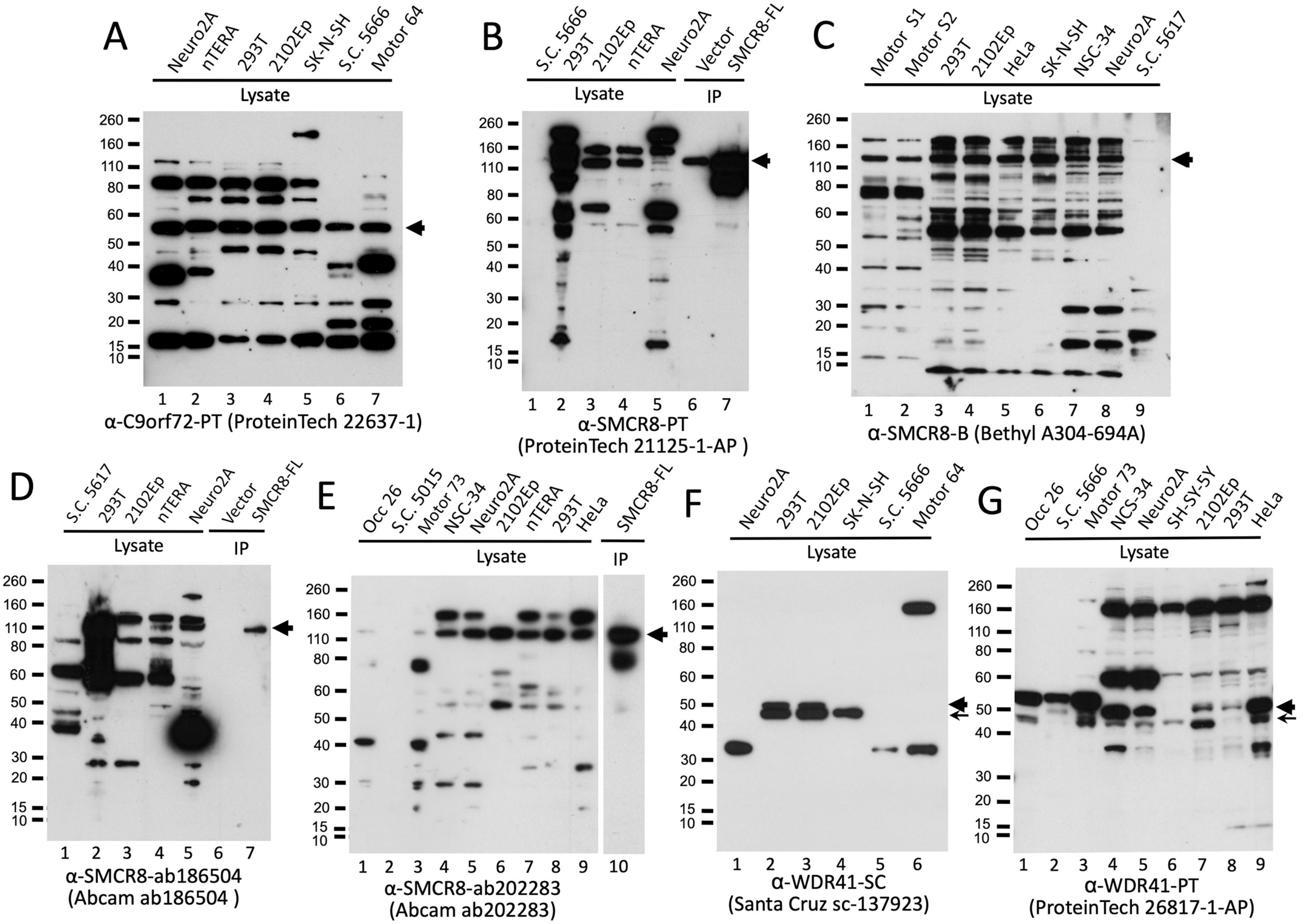
A
Neuro2A
nTERA
293T
2102Ep
SK-N-SH
S.C. 5666
Motor 64
Lysate
260
160
110
80
60
50
40
30
20
15
10
1 2 3 4 5 6 7
α-C9orf72-PT (ProteinTech 22637-1)
B
S.C. 5666
293T
2102Ep
nTERA
Neuro2A
Vector
SMCR8-FL
Lysate
IP
1 2 3 4 5 6 7
α-SMCR8-PT
(ProteinTech 21125-1-AP)
C
Motor S1
Motor S2
293T
2102Ep
HeLa
SK-N-SH
NSC-34
Neuro2A
S.C. 5617
Lysate
1 2 3 4 5 6 7 8 9
α-SMCR8-B (Bethyl A304-694A)
D
S.C. 5617
293T
2102Ep
nTERA
Neuro2A
Vector
SMCR8-FL
Lysate
IP
1 2 3 4 5 6 7
α-SMCR8-ab186504
(Abcam ab186504)
E
Occ 26
S.C. 5015
Motor 73
NSC-34
Neuro2A
2102Ep
nTERA
293T
HeLa
SMCR8-FL
Lysate
IP
1 2 3 4 5 6 7 8 9 10
α-SMCR8-ab202283
(Abcam ab202283)
F
Neuro2A
293T
2102Ep
SK-N-SH
S.C. 5666
Motor 64
Lysate
1 2 3 4 5 6
α-WDR41-SC
(Santa Cruz sc-137923)
G
Occ 26
S.C. 5666
Motor 73
NCS-34
Neuro2A
SH-SY-5Y
2102Ep
293T
HeLa
Lysate
1 2 3 4 5 6 7 8 9
α-WDR41-PT
(ProteinTech 26817-1-AP)

Figure S1

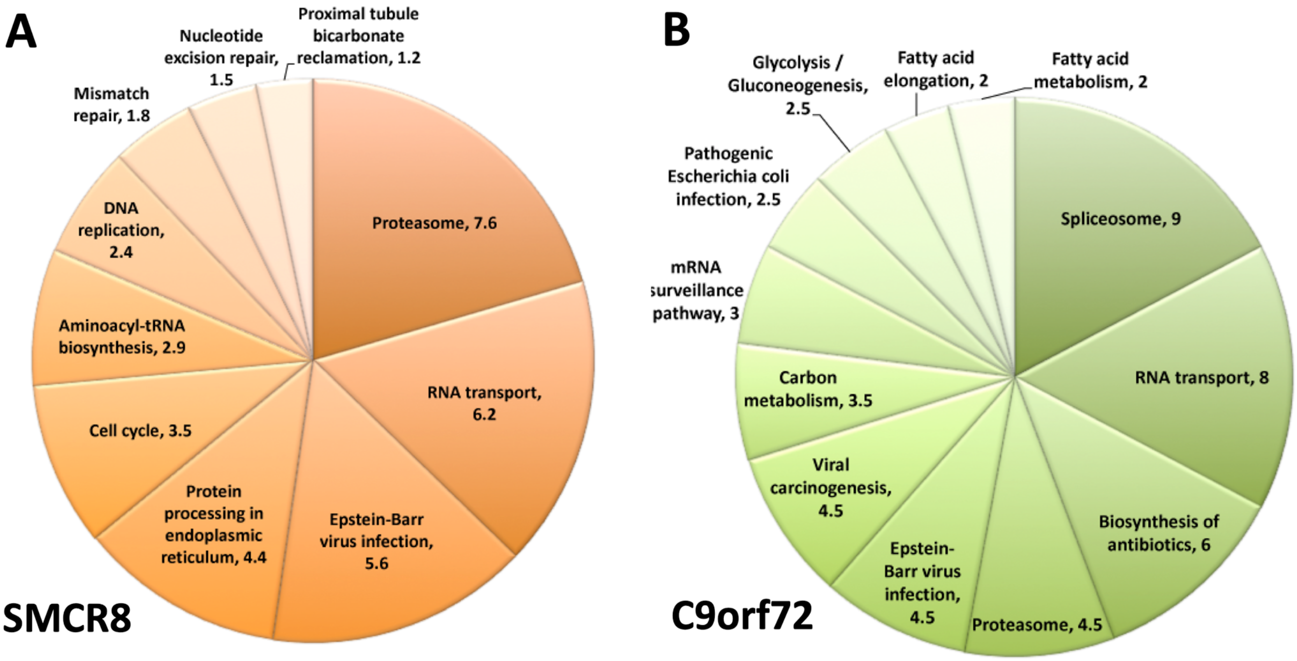
A
Proteasome, 7.6
RNA transport, 6.2
Epstein-Barr virus infection, 5.6
Protein processing in endoplasmic reticulum, 4.4
Cell cycle, 3.5
Aminoacyl-tRNA biosynthesis, 2.9
DNA replication, 2.4
Mismatch repair, 1.8
Nucleotide excision repair, 1.5
Proximal tubule bicarbonate reclamation, 1.2
SMCR8
B
Spliceosome, 9
RNA transport, 8
Biosynthesis of antibiotics, 6
Proteasome, 4.5
Epstein-Barr virus infection, 4.5
Viral carcinogenesis, 4.5
Carbon metabolism, 3.5
mRNA surveillance pathway, 3
Pathogenic Escherichia coli infection, 2.5
Glycolysis / Gluconeogenesis, 2.5
Fatty acid elongation, 2
Fatty acid metabolism, 2
C9orf72

Figure S2

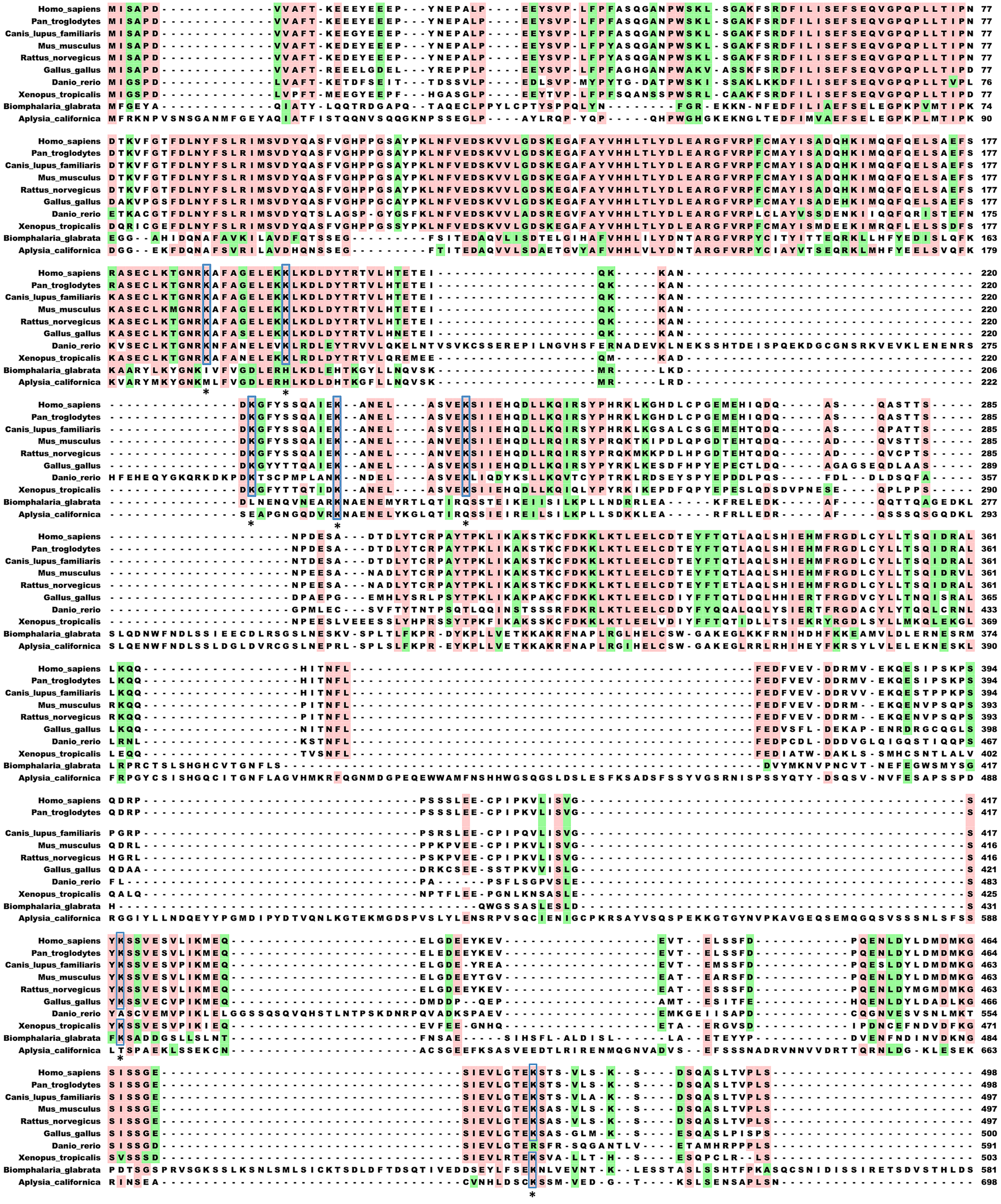

Figure S3-1

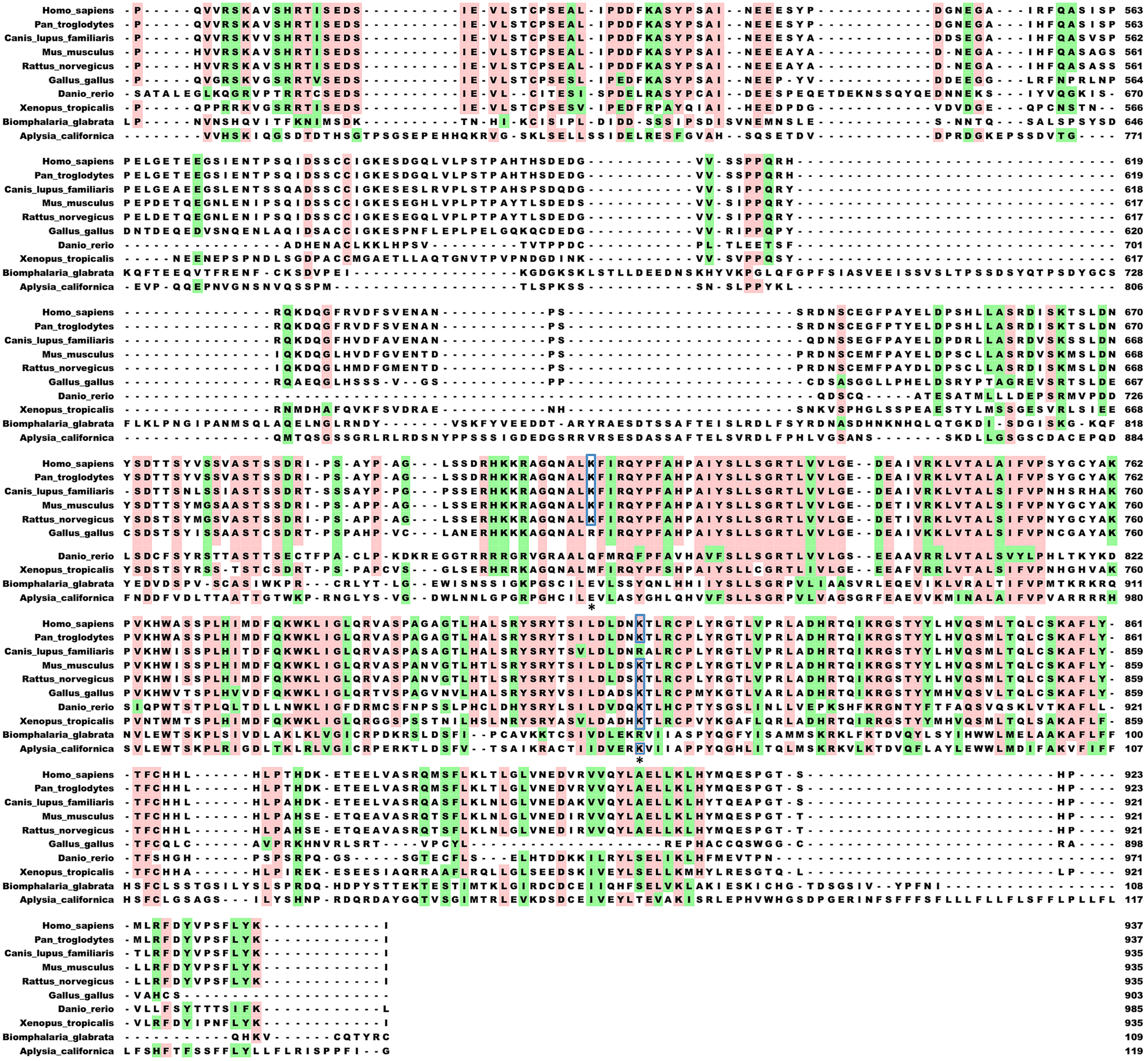

Figure S3-2

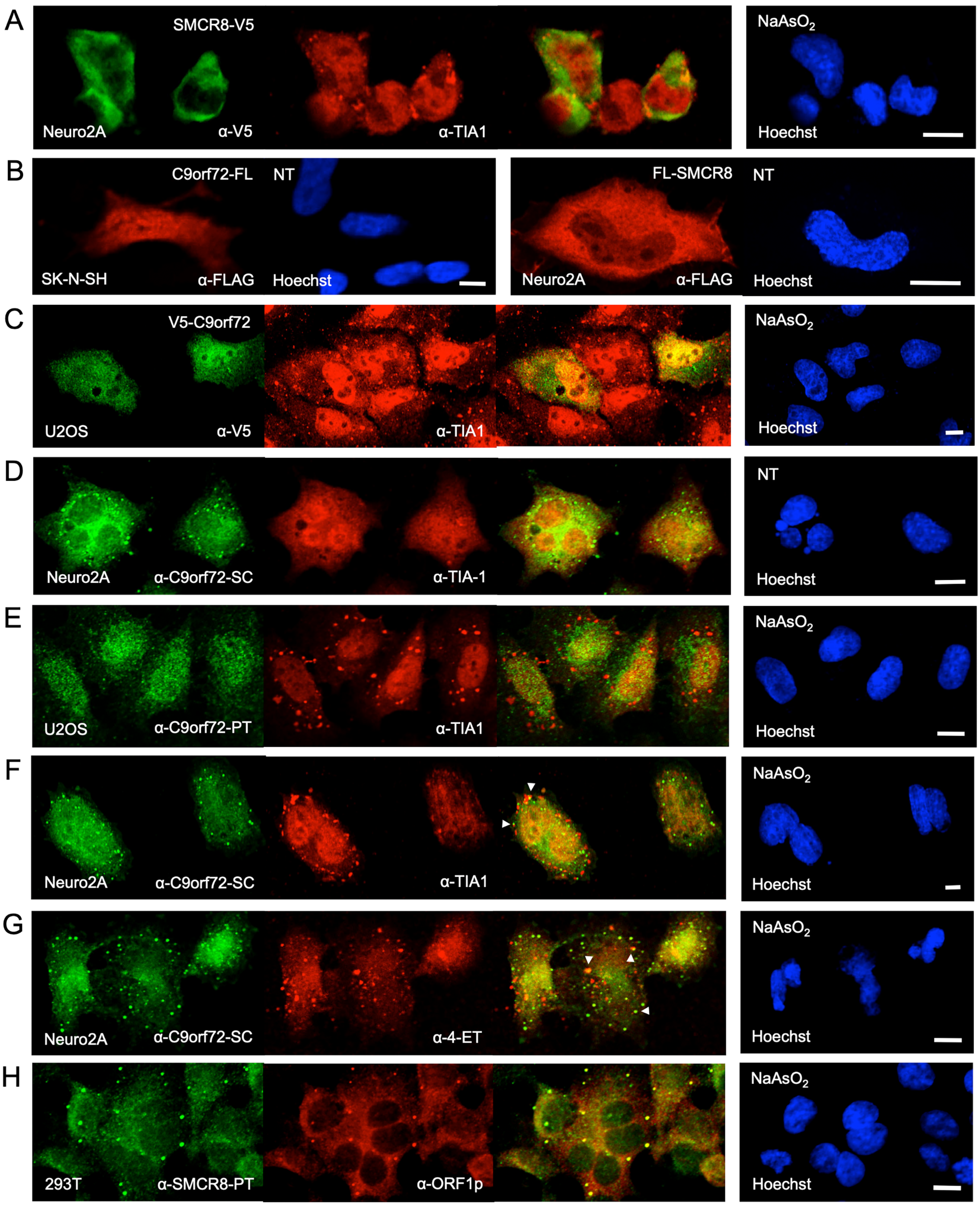
A
SMCR8-V5
Neuro2A
α-V5
α-TIA1
NaAsO2
Hoechst
B
C9orf72-FL
NT
SK-N-SH
α-FLAG
Hoechst
FL-SMCR8
NT
Neuro2A
α-FLAG
Hoechst
C
V5-C9orf72
U2OS
α-V5
α-TIA1
NaAsO2
Hoechst
D
Neuro2A
α-C9orf72-SC
α-TIA-1
NT
Hoechst
E
U2OS
α-C9orf72-PT
α-TIA1
NaAsO2
Hoechst
F
Neuro2A
α-C9orf72-SC
α-TIA1
NaAsO2
Hoechst
G
Neuro2A
α-C9orf72-SC
α-4-ET
NaAsO2
Hoechst
H
293T
α-SMCR8-PT
α-ORF1p
NaAsO2
Hoechst

Figure S4-1

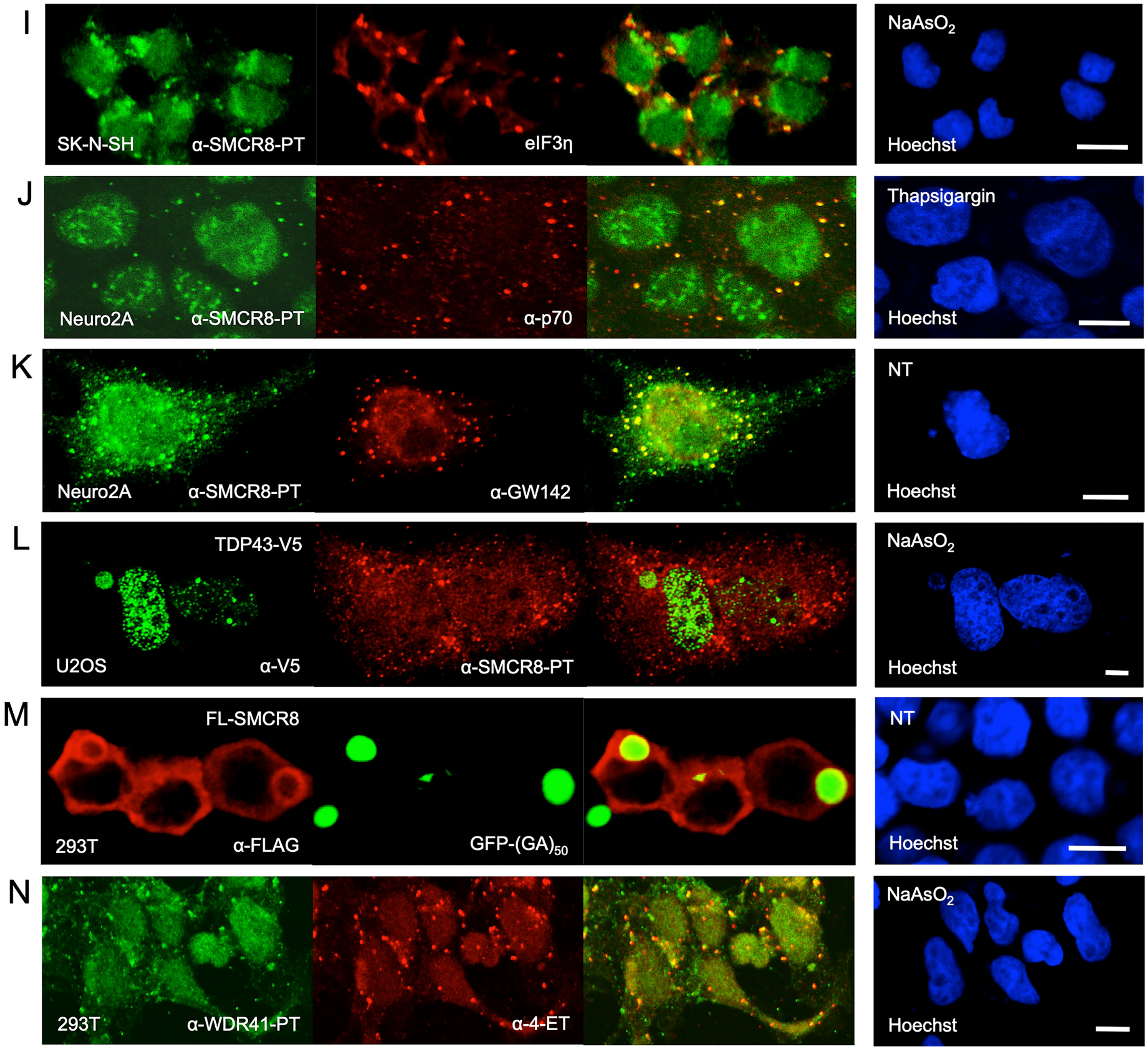
I
SK-N-SH
α-SMCR8-PT
eIF3η
NaAsO2
Hoechst
J
Neuro2A
α-SMCR8-PT
α-p70
Thapsigargin
Hoechst
K
Neuro2A
α-SMCR8-PT
α-GW142
NT
Hoechst
L
TDP43-V5
U2OS
α-V5
α-SMCR8-PT
NaAsO2
Hoechst
M
FL-SMCR8
293T
α-FLAG
GFP-(GA)50
NT
Hoechst
N
293T
α-WDR41-PT
α-4-ET
NaAsO2
Hoechst

Figure S4-2

Table S1. The C9orf72 protein interactome determined by mass spectrometry

Gene Name	Entrez Gene #	Protein Name	Accession Number	Molecular Weight (kD)	Total # Spectra		# Unique Peptides		# Unique Spectra		% Spectra		In C9orf72 interactome reported elsewhere [reference #]	In SMCR8 interactome (this study)	Identified in MGSP [102]
					RNase										
					-	+	-	+	-	+	-	+			
ACAA2	10449	acetyl-CoA acyltransferase 2	167614485	42	4	8	1	3	1	3	0.01	0.02			
ACOT7	11332	acyl-CoA thioesterase 7	32528282	42	2	4	1	2	1	2	0.01	0.01			
ACSL3	2181	acyl-CoA synthetase long-chain family member 3	42794752	80	2	4	1	2	1	2	0.01	0.01			
ACTA1	58	actin, alpha 1, skeletal muscle	4501881	42	0	6	0	1	0	2	0	0.02	51,88		
ACTN1	87	actinin alpha 1	194097350	106	0	4	0	2	0	2	0	0.01	51,88		
ACTR1A	10121	ARP1 actin-related protein 1 homolog A, centractin alpha	5031569	43	2	14	1	6	1	6	0.01	0.04			Yes
AIFM1	9131	apoptosis inducing factor, mitochondria associated 1	22202629	66	4	4	3	2	3	2	0.01	0.01		Yes	
AKAP17A	8227	A-kinase anchoring protein 17A	41152097	81	3	0	2	0	2	0	0.01	0			
ALYREF	10189	Aly/REF export factor	238776833	28	0	6	0	2	0	3	0	0.05	51,88		
AMY1A	276	amylase, alpha 1B	56549662	58	0	4	0	2	0	2	0	0.01		Yes	
ANXA1	301	annexin A1	767957250	39	11	2	5	2	5	2	0.02	0		Yes	Yes
AP2A2	161	adaptor related protein complex 2 alpha 2 subunit	27477041	104	4	0	2	0	2	0	0.01	0	51,88		
ARG1	383	arginase 1	10947139	35	6	10	2	6	3	6	0.01	0.02			
ATP2A2	488	ATPase sarcoplasmic/endoplasmic reticulum Ca2+ transporting 2	24638454	115	0	9	0	4	0	4	0	0.02	51,87	Yes	
ATP5A1	498	ATP synthase, H+ transporting, mitochondrial F1 complex, alpha subunit 1, cardiac muscle	382546190	54	2	4	1	2	1	2	0.01	0.01	51,88		
ATP5B	506	ATP synthase, H+ transporting, mitochondrial F1 complex, beta polypeptide	32189394	57	4	4	2	2	2	2	0.01	0.01	51		
AZGP1	563	alpha-2-glycoprotein 1, zinc-binding	4502337	34	3	16	2	4	2	4	0.01	0.03			
BCLAF1	9774	BCL2 associated transcription factor 1	117938251	100	7	0	1	0	3	0	0.02	0	51,88		
C9orf72	203228	chromosome 9 open reading frame 72	365906244	54	108	103	21	19	33	32	0.27	0.26		Yes	Yes
CALML5	51806	calmodulin like 5	223278387	16	20	0	2	0	5	0	0.04	0			Yes
CALU	813	calumenin	314122177	38	2	8	1	3	1	5	0.01	0.02		Yes	
CAND1	55832	cullin associated and neddylation dissociated 1	21361794	136	2	3	1	2	1	2	0.01	0.01	51		
CAPZA1	829	capping actin protein of muscle Z-line alpha subunit 1	5453597	33	0	4	0	2	0	2	0	0.01	51,88		
CAT	847	catalase	4557014	60	0	3	0	2	0	2	0	0.01	51		
CCAR1	55749	cell division cycle and apoptosis regulator 1	46852388	133	4	6	2	3	2	3	0.01	0.02			Yes
CD2BP2	10421	CD2 cytoplasmic tail binding protein 2	343887347	38	4	6	2	3	2	3	0.01	0.02			
CDC42	998	cell division cycle 42	4757952	21	0	4	0	1	0	2	0	0.01	51		
CDC73	79577	cell division cycle 73	578801489	39	0	4	0	2	0	2	0	0.01		Yes	Yes
CDK1	983	cyclin dependent kinase 1	4502709	34	4	4	2	2	2	2	0.01	0.01	51		Yes
CDSN	1041	corneodesmosin	67782356	52	17	8	4	2	5	2	0.03	0.02			
CEP350	9857	centrosomal protein 350	171184451	351	0	4	0	2	0	2	0	0.01			
CHTOP	26097	chromatin target of PRMT1	28875797	26	8	0	2	0	2	0	0.02	0		Yes	
COPB1	1315	coatomer protein complex subunit beta 1	221316630	107	14	14	6	5	7	5	0.04	0.04		Yes	
CORO1B	57175	coronin 1B	14149734	54	0	3	0	2	0	2	0	0.01	51		Yes
CPSF1	29894	cleavage and polyadenylation specific factor 1	56676371	161	4	2	2	1	2	1	0.01	0.01			
CPSF2	53981	cleavage and polyadenylation specific factor 2	530403998	88	6	0	2	0	3	0	0.01	0		Yes	
CSNK2A1	1457	casein kinase 2 alpha 1	29570791	45	3	3	2	2	2	2	0.01	0.01	51		
CTSD	1509	cathepsin D	4503143	45	0	9	0	3	0	3	0	0.02		Yes	
DCTN2	10540	dynactin subunit 2	387527974	44	0	5	0	3	0	4	0	0.01	29,51	Yes	
DDB1	1642	damage specific DNA binding protein 1	148529014	127	6	4	2	2	3	2	0.02	0.01	51	Yes	
DDX20	11218	DEAD-box helicase 20	256223453	92	0	4	0	2	0	2	0	0.01			
DDX46	9879	DEAD-box helicase 46	664806097	117	6	8	2	3	2	3	0.02	0.02			
DENND5A	23258	DENN domain containing 5A	343098489	142	1	6	1	1	1	2	0.01	0.05			
DIS3	22894	DIS3 homolog, exosome endoribonuclease and 3'-5' exoribonuclease	190014623	109	6	12	3	5	3	5	0.02	0.03		Yes	
DNAJA1	3301	DnaJ heat shock protein family member A1	4504511	45	4	2	2	1	2	1	0.03	0.02	28,51,87,88	Yes	Yes
DNAJC13	23317	DnaJ heat shock protein family member C13	112421122	254	4	0	2	0	2	0	0.01	0			
DPM1	8813	dolichyl-phosphate mannosyltransferase subunit 1, catalytic	4503363	30	3	0	2	0	2	0	0.01	0		Yes	
DSC1	1823	desmocollin 1	13435361	100	14	15	4	3	6	4	0.03	0.03		Yes	
DSC3	1825	desmocollin 3	148539846	100	4	0	3	0	3	0	0.01	0			
EEF2	1938	eukaryotic translation elongation factor 2	4503483	95	15	18	8	8	8	8	0.04	0.05	51,87,88		
EIF2S1	1965	eukaryotic translation initiation factor 2 subunit alpha	4758256	36	0	4	0	2	0	2	0	0.01	51		
EIF2S3	1968	eukaryotic translation initiation factor 2 subunit gamma	4503507	51	12	10	5	5	6	5	0.03	0.03	88		
EIF4G2	1982	eukaryotic translation initiation factor 4 gamma 2	289577080	102	6	12	3	5	3	5	0.02	0.03			Yes
ERH	2079	enhancer of rudimentary homolog	4758302	12	5	0	3	0	3	0	0.01	0	51		
FABP5	2171	fatty acid binding protein 5	4557581	15	15	0	3	0	3	0	0.03	0			
FLG2	388698	filaggrin family member 2	62122917	248	33	6	10	3	15	3	0.06	0.01			
FLII	2314	FLII, actin remodeling protein	372266127	144	2	4	1	2	1	2	0.01	0.01	51,88		
FMR1	2332	fragile X mental retardation 1	297374779	69	4	0	2	0	2	0	0.01	0		Yes	
GALK1	2584	galactokinase 1	4503895	42	0	4	0	2	0	2	0	0.01		Yes	
GAPDH	2597	glyceraldehyde-3-phosphate dehydrogenase	378404908	32	4	4	2	2	2	2	0.03	0.03	51,88		
GCN1	10985	GCN1, eIF2 alpha kinase activator homolog	54607053	293	10	8	5	3	5	3	0.03	0.02			
GNL3	26354	G protein nucleolar 3	45593130	62	4	0	2	0	2	0	0.01	0			
GTF2B	2959	general transcription factor IIB	4504193	35	0	4	0	2	0	2	0	0.01			
GTF2I	2969	general transcription factor IIi	14670350	112	0	3	0	3	0	3	0	0.01	51	Yes	
HADHA	3030	hydroxyacyl-CoA dehydrogenase/3-ketoacyl-CoA thiolase/enoyl-CoA hydratase , alpha subunit	20127408	83	18	14	7	7	9	7	0.05	0.04	51,88	Yes	
HBA1	3039	hemoglobin subunit alpha 2	4504345	15	0	28	0	5	0	8	0	0.07			
HBB	3043	hemoglobin subunit beta	4504349	16	0	26	0	7	0	8	0	0.07			
HDAC1	3065	histone deacetylase 1	13128860	55	2	4	1	2	1	2	0.01	0.01	51		
HIST1H1C	3006	histone cluster 1 H1 family member c	4885375	21	2	6	1	2	1	2	0.02	0.05	88		
HK1	3098	hexokinase 1	15991827	102	6	2	2	1	2	1	0.02	0.01			
HK2	3099	hexokinase 2	15553127	102	0	6	0	3	0	3	0	0.02	51		
HNRNPA3	220988	heterogeneous nuclear ribonucleoprotein A3	530369990	40	9	0	2	0	2	0	0.02	0	51,88	Yes	Yes
HNRNPD	3184	heterogeneous nuclear ribonucleoprotein D	14110414	33	6	0	2	0	2	0	0.01	0	51		Yes
HNRNPH2	3188	heterogeneous nuclear ribonucleoprotein H2	74099697	49	12	9	3	2	4	3	0.03	0.02	88	Yes	Yes
HNRNPM	4670	heterogeneous nuclear ribonucleoprotein M	530427503	76	0	14	0	6	0	7	0	0.11	51,88	Yes	
HNRNPU	3192	heterogeneous nuclear ribonucleoprotein U	14141161	89	0	7	0	4	0	4	0	0.06	51,88		
HUWE1	10075	HECT, UBA and WWE domain containing 1, E3 ubiquitin protein ligase	530426344	482	3	10	2	5	2	5	0.01	0.03	29	Yes	
IARS	3376	isoleucyl-tRNA synthetase	94721239	145	21	22	10	10	11	11	0.05	0.06	51	Yes	
IDE	3416	insulin degrading enzyme	155969707	118	3	0	2	0	2	0	0.01	0			
KCTD5	54442	potassium channel tetramerization domain containing 5	9506651	26	6	0	2	0	2	0	0.02	0			
KHDRBS1	10657	KH RNA binding domain containing, signal transduction associated 1	5730027	48	12	6	3	3	3	3	0.03	0.02	51,88		Yes
KPNA2	3838	karyopherin subunit alpha 2	4504897	58	4	4	2	2	2	2	0.01	0.01	88		Yes
KPNB1	3837	karyopherin subunit beta 1	19923142	97	5	8	3	4	3	4	0.01	0.02	19,51,88	Yes	Yes
LARS	51520	leucyl-tRNA synthetase	108773810	134	8	25	4	11	4	12	0.02	0.06	51	Yes	
LCN1	3933	lipocalin 1	4504963	19	0	8	0	2	0	2	0	0.02			
LGALS7	3963	galectin 7B	109948279	15	8	0	2	0	2	0	0.02	0			
LIMA1	51474	LIM domain and actin binding 1	165905589	85	2	4	1	2	1	2	0.01	0.01	51,88		
LMNA	4000	lamin A/C	383792150	64	4	0	2	0	2	0	0.01	0	88	Yes	Yes
LOC441155	441155	zinc finger CCCH-type domain-containing-like	410812199	89	2	4	1	1	1	1	0.01	0.01			
LRPPRC	10128	leucine rich pentatricopeptide repeat containing	767913641	155	23	25	11	11	11	12	0.06	0.06		Yes	
LTF	4057	lactotransferrin	312433998	73	0	13	0	6	0	7	0	0.03			
LUC7L3	51747	LUC7 like 3 pre-mRNA splicing factor	52426743	51	13	4	2	2	4	2	0.02	0.01	51	Yes	
MAGED2	10916	MAGE family member D2	29171705	65	5	0	2	0	2	0	0.01	0		Yes	Yes
MAGOH	4116	mago homolog, exon junction complex core component	4505087	17	8	0	3	0	3	0	0.02	0			
MAP7	9053	microtubule associated protein 7	310750348	87	0	4	0	2	0	2	0	0.01	88		
MARS	4141	methionyl-tRNA synthetase	14043022	101	0	3	0	2	0	2	0	0.01	51	Yes	Yes
MFAP1	4236	microfibrillar associated protein 1	50726968	52	4	2	2	1	2	1	0.01	0.01			Yes
MLLT11	10962	Super Elongation Complex Subunit	5802968	10	4	1	2	1	2	1	0.01	0			
MSH2	4436	mutS homolog 2	384871702	97	0	4	0	2	0	2	0	0.01			
MYBBP1A	10514	MYB binding protein 1a	157694492	149	7	0	4	0	4	0	0.02	0	51		
MYO1D	4642	myosin ID	51100974	116	4	0	2	0	2	0	0.01	0			
NAP1L1	4673	nucleosome assembly protein 1 like 1	21327708	45	0	4	0	2	0	2	0	0.03	51,88		
NCBP1	4686	nuclear cap binding protein subunit 1	767957513	92	4	0	2	0	2	0	0.01	0			
NME1	4830	NME/NM23 nucleoside diphosphate kinase 1	38045913	20	1	4	1	2	1	2	0	0.01	51		
NPM1	4869	nucleophosmin	10835063	33	0	15	0	4	0	7	0	0.12	51,88		
NPM3	10360	nucleophosmin/nucleoplasmin 3	6857818	19	1	4	1	1	1	1	0	0.01	51		
NUFIP2	57532	NUFIP2, FMR1 interacting protein 2	32698730	76	0	4	0	2	0	2	0	0.01			Yes
NUP133	55746	nucleoporin 133	26051235	129	0	4	0	2	0	2	0	0.01		Yes	
NUP155	9631	nucleoporin 155	24430149	155	2	4	1	2	1	2	0.01	0.01	51	Yes	
NUP93	9688	nucleoporin 93	208609990	93	8	16	4	7	4	7	0.02	0.04		Yes	
OAT	4942	ornithine aminotransferase	4557809	49	4	8	2	5	2	5	0.01	0.02			

PA2G4	5036	proliferation-associated 2G4	124494254	44	4	10	2	3	2	3	0.01	0.03			Yes
PCGF6	84108	polycomb group ring finger 6	58761530	39	0	4	0	2	0	2	0	0.01			
PDHB	5162	pyruvate dehydrogenase beta	156564403	39	4	11	2	6	2	6	0.01	0.03	87		
PDIA6	10130	protein disulfide isomerase family A member 6	544186032	54	8	8	4	4	4	4	0.02	0.02	51		
PDXP	57026	pyridoxal phosphatase	10092677	32	0	6	0	3	0	3	0	0.02			
PFAS	5198	phosphoribosylformylglycinamidine synthase	31657129	145	6	10	3	5	3	5	0.02	0.03			
PIP	5304	prolactin induced protein	4505821	17	0	4	0	2	0	2	0	0.03			
PKP1	5317	plakophilin 1	53729344	80	6	7	3	4	3	4	0.01	0.02			Yes
PNP	4860	purine nucleoside phosphorylase	157168362	32	7	0	4	0	4	0	0.01	0	51		
POF1B	79983	premature ovarian failure, 1B	193794853	68	3	0	2	0	2	0	0.01	0			
POLD1	5424	DNA polymerase delta 1, catalytic subunit	156616275	124	2	4	1	2	1	2	0.01	0.01		Yes	
POLR1C	9533	RNA polymerase I polypeptide C	42560246	39	0	3	0	1	0	2	0	0.01			
PPM1F	9647	protein phosphatase, Mg2+/Mn2+ dependent 1F	7661862	50	0	4	0	2	0	2	0	0.01			
PRDX6	9588	peroxiredoxin 6	4758638	25	5	4	2	2	3	2	0.01	0.01	51	Yes	Yes
PRPF31	26121	pre-mRNA processing factor 31	221136939	55	0	4	0	2	0	2	0	0.03			
PRPSAP2	5636	phosphoribosyl pyrophosphate synthetase associated protein 2	345478663	31	7	12	3	4	4	5	0.02	0.03			
PSMA6	5687	proteasome subunit alpha 6	23110944	27	0	3	0	2	0	2	0	0.01	51	Yes	
PSMC1	5700	proteasome 26S subunit, ATPase 1	24430151	49	5	8	3	4	3	4	0.01	0.02	51	Yes	
PSMC2	5701	proteasome 26S subunit, ATPase 2	4506209	49	12	25	5	10	6	12	0.03	0.06	51	Yes	
PSMC3	5702	proteasome 26S subunit, ATPase 3	21361144	49	6	16	3	6	3	7	0.02	0.04	51	Yes	
PSMC6	5706	proteasome 26S subunit, ATPase 6	195539395	46	5	6	3	3	3	3	0.01	0.02	51	Yes	
PSMD1	5707	proteasome 26S subunit, non-ATPase 1	25777600	106	2	3	1	2	1	2	0.01	0.01	51	Yes	
PSMD14	10213	proteasome 26S subunit, non-ATPase 14	5031981	35	2	4	1	2	1	2	0.01	0.01	51	Yes	
PSMD3	5709	proteasome 26S subunit, non-ATPase 3	25777612	61	2	9	1	4	1	5	0.01	0.02	51	Yes	
PSMD4	5710	proteasome 26S subunit, non-ATPase 4	5292161	41	0	10	0	4	0	4	0	0.03	29,51		
RAB1B	81876	RAB1B, member RAS oncogene family	13569962	22	5	0	2	0	2	0	0.01	0	51	Yes	
RANBP2	5903	RAN binding protein 2	150418007	358	4	16	2	7	2	7	0.01	0.04		Yes	
RARS	5917	arginyl-tRNA synthetase	15149476	75	6	4	3	2	3	2	0.02	0.01	51	Yes	
RB1CC1	9821	RB1 inducible coiled-coil 1	134304846	183	8	6	4	3	4	3	0.02	0.01	29	Yes	
RBBP7	5931	RB binding protein 7, chromatin remodeling factor	4506439	48	0	4	0	1	0	1	0	0.01	51	Yes	
RBM15	64783	RNA binding motif protein 15	319996623	106	4	0	2	0	2	0	0.01	0			
RBM22	55696	RNA binding motif protein 22	8922328	47	0	5	0	3	0	3	0	0.01			
RBM6	10180	RNA binding motif protein 6	5032033	129	1	4	1	1	1	2	0	0.01			
RBM8A	9939	RNA binding motif protein 8A	4826972	20	5	0	2	0	3	0	0.01	0			
RCL1	10171	RNA terminal phosphate cyclase like 1	157426877	41	4	0	2	0	2	0	0.01	0			
RCN1	5954	reticulocalbin 1	4506455	39	0	8	0	3	0	3	0	0.02			
RCN2	5955	reticulocalbin 2	426214088	39	0	4	0	2	0	2	0	0.03	51	Yes	
RHOA	387	ras homolog family member A	10835049	22	0	4	0	2	0	2	0	0.01	51		Yes
RIC8A	60626	RIC8 guanine nucleotide exchange factor A	530395630	60	0	4	0	2	0	2	0	0.01			
RNF2	6045	ring finger protein 2	767910100	38	3	0	2	0	2	0	0.01	0	51	Yes	
ROCK1	6093	Rho associated coiled-coil containing protein kinase 1	4885583	158	2	4	1	1	1	2	0.01	0.01			Yes
RUVBL1	8607	RuvB like AAA ATPase 1	4506753	50	6	12	2	5	2	5	0.02	0.03	51,88		
S100A8	6279	S100 calcium binding protein A8	21614544	11	0	3	0	1	0	1	0	0.01			Yes
S100A9	6280	S100 calcium binding protein A9	4506773	13	8	6	3	3	5	5	0.02	0.01			Yes
SERBP1	26135	SERPINE1 mRNA binding protein 1	66346683	43	0	7	0	4	0	4	0	0.06	51,88		
SERPINA1	5265	serpin family A member 1	50363217	47	0	5	0	2	0	3	0	0.01			
SERPINB3	6317	serpin family B member 3	5902072	45	21	0	7	0	10	0	0.04	0		Yes	
SF3B1	23451	splicing factor 3b subunit 1	530370087	130	0	6	0	3	0	3	0	0.05	51		
SF3B2	10992	splicing factor 3b subunit 2	530396224	100	0	6	0	3	0	3	0	0.05	51		
SF3B4	10262	splicing factor 3b subunit 4	5032069	44	2	3	1	2	1	2	0.01	0.01			
SFN	2810	stratifin	5454052	28	0	4	0	1	0	1	0	0.01	88		Yes
SFPQ	6421	splicing factor proline and glutamine rich	4826998	76	4	10	2	3	2	4	0.01	0.03	51		Yes
SFXN1	94081	sideroflexin 1	23618867	36	2	4	1	2	1	2	0.01	0.01		Yes	
SLC25A11	8402	solute carrier family 25 member 11	21361114	34	4	0	2	0	2	0	0.01	0	28,32,88	Yes	
SLC25A3	5250	solute carrier family 25 member 3	47132595	40	7	9	2	2	3	3	0.01	0.02	28,51,87,88	Yes	
SLC25A6	293	solute carrier family 25 member 6	156071462	33	9	4	2	2	3	2	0.02	0.01	32,88	Yes	
SLIRP	81892	SRA stem-loop interacting RNA binding protein	13654278	12	0	4	0	2	0	2	0	0.01			
SLITRK5	26050	SLIT and NTRK like family member 5	530423328	107	0	4	0	2	0	2	0	0.01			
SMC1A	8243	structural maintenance of chromosomes 1A	30581135	143	0	8	0	4	0	4	0	0.02		Yes	
SMCR8	140775	Smith-Magenis syndrome chromosome region, candidate 8	146260268	105	165	217	40	43	54	59	0.41	0.56	28,29,32,87	Yes	
SNRPD3	6634	small nuclear ribonucleoprotein D3 polypeptide	4759160	14	2	4	1	2	1	2	0.02	0.03	51		
SNRPE	6635	small nuclear ribonucleoprotein polypeptide E	4507129	11	5	4	2	2	3	2	0.01	0.01		Yes	
SPIN2B	474343	spindlin family member 2B	544315535	18	2	12	1	4	1	5	0.01	0.03			
SRP72	6731	signal recognition particle 72	109638749	75	0	4	0	2	0	2	0	0.01	51		
SSRP1	6749	structure specific recognition protein 1	4507241	81	4	4	1	1	1	1	0.01	0.01		Yes	
STOML2	30968	stomatin like 2	559098406	33	0	6	0	3	0	3	0	0.02		Yes	
SUGT1	10910	SGT1 homolog, MIS12 kinetochore complex assembly cochaperone	195963398	41	6	8	2	4	3	4	0.02	0.02		Yes	
SUMO3	6612	small ubiquitin-like modifier 3	48928058	12	0	3	0	1	0	1	0	0.01			
SYNCRIP	10492	synaptotagmin binding cytoplasmic RNA interacting protein	228008291	70	6	2	2	1	2	1	0.02	0.01	88		
TAF15	8148	TATA-box binding protein associated factor 15	21327701	62	0	4	0	2	0	2	0	0.01	88		Yes
TECR	9524	trans-2,3-enoyl-CoA reductase	24475816	36	0	3	0	2	0	2	0	0.01	51	Yes	
TF	7018	transferrin	4557871	77	0	5	0	3	0	3	0	0.01			
THOC3	84321	THO complex 3	14150171	39	2	4	1	2	1	2	0.01	0.01		Yes	
TIAL1	7073	TIA1 cytotoxic granule associated RNA binding protein like 1	77695912	43	6	2	3	1	3	1	0.02	0.01			
TIMM50	92609	translocase of inner mitochondrial membrane 50	48526509	50	3	0	2	0	2	0	0.01	0	51,87	Yes	
TMOD3	29766	tropomodulin 3	7657649	40	0	6	0	2	0	3	0	0.02	51		Yes
TOP1	7150	topoisomerase I	11225260	91	3	0	3	0	3	0	0.01	0	51		
TPI1	7167	triosephosphate isomerase 1	226529917	31	3	0	2	0	2	0	0.01	0	51		
TROVE2	6738	TROVE domain family member 2	108796056	59	0	3	0	2	0	2	0	0.01			
TUBB2A	7280	tubulin beta 2A class IIa	4507729	50	0	4	0	2	0	2	0	0.03	32,51,B7,88		
TUBB3	10381	tubulin beta 3 class III	50592996	50	2	4	1	2	1	2	0.01	0.01	51,88		Yes
TXN	7295	thioredoxin	50592994	12	7	0	2	0	2	0	0.01	0	51	Yes	Yes
U2AF2	11338	U2 small nuclear RNA auxiliary factor 2	6005926	54	14	6	4	2	7	2	0.04	0.02	51	Yes	
U2SURP	23350	U2 snRNP associated SURP domain containing	122937227	118	0	3	0	2	0	2	0	0.02			
UBB	7314	ubiquitin B	11024714	26	2	4	2	2	2	2	0.02	0.03	88		
UBR4	23352	ubiquitin protein ligase E3 component n-recognin 4	767903406	579	2	4	1	2	1	2	0.01	0.01	29		
WDHD1	11169	WD repeat and HMG-box DNA binding protein 1	56550086	113	0	4	0	2	0	2	0	0.01			
WDR41	55255	WD repeat domain 41	42716287	52	69	85	20	19	27	28	0.17	0.22	28.,29,32,87	Yes	
WTAP	9589	Wilms tumor 1 associated protein	21361159	44	4	0	2	0	2	0	0.01	0			
YBX1	4904	Y-box binding protein 1	34098946	36	0	8	0	4	0	4	0	0.06	51,88		Yes

Table S2. The SMCR8 protein interactome determined by mass spectrometry

Gene Name	Entrez Gene #	Protein Name	Accession Number	Molecular Weight (kD)	Total # Spectra		# Unique Peptides		# Unique Spectra		% Spectra		In C9orf72 interactome (this study)	In C9 interactome reported elsewhere [reference #]	Identified in [34]	Identified in MGSP [102]
					RNase											
					-	+	-	+	-	+	-	+				
AASDHPPT	4860	aminoadipate-semialdehyde dehydrogenase-phosphopantetheinyl transferase	20357568	36	3	0	2	0	2	0	0.01	0.00				
ABCF2	10061	ATP binding cassette subfamily F member 2	27881506	71	0	3	0	2	0	2	0.00	0.01		51	Yes	
ACAT1	38	acetyl-CoA acetyltransferase 1	4557237	45	5	12	5	5	5	5	0.01	0.02		51	Yes	
ACBD3	64746	acyl-CoA binding domain containing 3	15826852	61	3	6	2	2	2	2	0.01	0.01				
ACP1	52	acid phosphatase 1, soluble	4757714	18	4	8	2	2	2	2	0.01	0.02		51	Yes	
ADAR	103	adenosine deaminase, RNA specific	578800406	137	6	2	4	2	4	2	0.01	0.00			Yes	
ADNP	23394	activity dependent neuroprotector homeobox	768016782	123	7	8	4	5	4	5	0.01	0.02				
AIFM1	9131	apoptosis inducing factor, mitochondria associated 1	22202629	66	18	18	8	8	8	8	0.03	0.03	Yes		Yes	
AIMP1	9255	aminoacyl tRNA synthetase complex interacting multifunctional protein 1	215490011	37	3	0	2	0	2	0	0.01	0.00		51	Yes	
AIMP2	7965	aminoacyl tRNA synthetase complex interacting multifunctional protein 2	11125770	35	3	2	2	2	2	2	0.01	0.00		51	Yes	
AIP	196	aryl hydrocarbon receptor interacting protein	729042247	38	14	13	5	5	7	7	0.03	0.03			Yes	
AKAP8	10270	A-kinase anchoring protein 8	5031579	76	4	0	2	0	2	0	0.01	0.00			Yes	
AKAP8L	26993	A-kinase anchoring protein 8 like	768001947	72	6	5	4	2	4	2	0.01	0.01			Yes	
ALDH18A1	5832	aldehyde dehydrogenase 18 family member A1	578819671	87	9	0	5	0	5	0	0.02	0.00			Yes	Yes
ALDH3A2	224	aldehyde dehydrogenase 3 family member A2	4557303	55	0	3	0	2	0	2	0.00	0.01			Yes	
AMBRA1	55626	autophagy and beclin 1 regulator 1	392306969	143	0	4	0	3	0	3	0.00	0.01		29	Yes	
AMY1A	276	amylase, alpha 1B	56549662	58	3	0	2	0	2	0	0.01	0.00	Yes			
ANKHD1-EIF4EBP3	54882	ANKHD1-EIF4EBP3 readthrough	37620163	277	7	5	3	3	3	3	0.01	0.01				
ANKRD28	23243	ankyrin repeat domain 28	530372158	116	5	5	4	3	4	3	0.01	0.01			Yes	
ANXA1	301	annexin A1	767957250	39	6	0	4	0	4	0	0.01	0.00	Yes			
AP3M1	26985	adaptor Related Protein Complex 3 Mu 1 Subunit	767962478	47	3	0	2	0	2	0	0.01	0.00				
ARCN1	372	archain 1	11863154	57	4	4	2	2	2	2	0.01	0.01			Yes	
ARF1	375	ADP ribosylation factor 1	4502201	21	0	4	0	2	0	2	0.00	0.01		51		
ARHGAP17	55114	Rho GTPase activating protein 17	54860079	95	5	5	2	4	2	4	0.01	0.01				
ARHGAP21	57584	Rho GTPase activating protein 21	530392343	217	0	4	0	2	0	2	0.00	0.01				
ARHGEF2	9181	Rho/Rac guanine nucleotide exchange factor 2	15011974	108	4	5	3	3	3	3	0.01	0.01		51		
ARL1	400	ADP ribosylation factor like GTPase 1	4502227	20	0	6	0	2	0	2	0.00	0.01		51	Yes	
ATAD3A	55210	ATPase family, AAA domain containing 3A	283436220	71	2	4	1	2	1	2	0.02	0.04		51	Yes	Yes
ATP1A1	476	ATPase Na+/K+ transporting subunit alpha 1	21361181	113	38	46	13	15	14	15	0.07	0.09		51	Yes	
ATP1B3	483	ATPase Na+/K+ transporting subunit beta 3	4502281	32	0	6	0	2	0	2	0.00	0.01			Yes	
ATP2A2	488	ATPase sarcoplasmic/endoplasmic reticulum Ca2+ transporting 2	24638454	115	19	22	8	8	8	8	0.04	0.04	Yes	51	Yes	
ATP5C1	509	ATP synthase, H+ transporting, mitochondrial F1 complex, gamma polypeptide 1	50345988	33	14	7	5	4	7	4	0.03	0.01		51	Yes	
ATP5O	539	ATP synthase, H+ transporting, mitochondrial F1 complex, O subunit	4502303	23	3	0	2	0	2	0	0.01	0.00		51	Yes	
ATXN2	6311	ataxin 2	171543895	140	4	0	2	0	2	0	0.01	0.00			Yes	
AURKB	9212	aurora kinase B	548960892	39	0	3	0	2	0	2	0.00	0.01				
BAG2	9532	BCL2 associated athanogene 2	4757834	24	11	16	3	5	4	5	0.02	0.03		51	Yes	
BAG3	9531	BCL2 associated athanogene 3	14043024	62	2	3	2	2	2	2	0.00	0.01		29,51	Yes	Yes
BAG5	9529	BCL2 associated athanogene 5	62548854	51	18	11	5	5	5	5	0.03	0.02			Yes	
BAG6	7917	BCL2 associated athanogene 6	149158692	119	11	8	4	3	4	3	0.02	0.02			Yes	
BSG	682	basigin	38372919	42	2	4	2	3	2	3	0.00	0.01		51	Yes	
C9ORF72	203228	chromosome 9 open reading frame 72	365906244	54	15	18	4	4	4	4	0.03	0.03	Yes	28,31,32	Yes	Yes
CACYBP	27101	calcyclin binding protein	60218913	21	9	4	4	2	4	2	0.02	0.01		51	Yes	
CAD	790	carbamoyl-phosphate synthetase 2, aspartate transcarbamylase, and dihydroorotase	18105007	243	62	48	25	20	27	21	0.11	0.09		51	Yes	
CALU	813	calumenin	314122177	38	5	16	2	7	3	9	0.01	0.03	Yes			
CARM1	10498	coactivator associated arginine methyltransferase 1	40288288	66	0	4	0	3	0	3	0.00	0.01		51	Yes	
CBWD2	150472	COBW domain containing 2	33469141	44	0	4	0	3	0	3	0.00	0.01			Yes	
CCNT1	904	cyclin T1	17978466	81	3	0	2	0	2	0	0.01	0.00				
CDC42EP1	11135	CDC42 effector protein 1	578836868	40	3	0	2	0	2	0	0.01	0.00			Yes	
CDC7	8317	cell division cycle 7	530363409	64	3	0	2	0	2	0	0.01	0.00			Yes	
CDC73	79577	cell division cycle 73	40018640	61	7	13	5	6	5	6	0.01	0.03	Yes			Yes
CDIPT	10423	CDP-diacylglycerol--inositol 3-phosphatidyltransferase	530407452	26	4	0	2	0	2	0	0.01	0.00				
CDK4	1019	cyclin dependent kinase 4	4502735	34	5	0	2	0	2	0	0.01	0.00		51	Yes	
CDK5	1020	cyclin dependent kinase 5	4826675	33	7	0	4	0	4	0	0.01	0.00			Yes	
CDKN2A	1029	cyclin dependent kinase inhibitor 2A	304376272	18	0	3	0	2	0	2	0.00	0.01			Yes	
CEP170	9859	centrosomal protein 170	109255228	175	6	5	4	3	4	3	0.01	0.01		51	Yes	
CHD4	1108	chromodomain helicase DNA binding protein 4	51599156	218	13	28	9	8	9	9	0.02	0.05				
CHTF18	63922	chromosome transmission fidelity factor 18	27501458	107	3	0	2	0	2	0	0.01	0.00			Yes	
CHTOP	26097	chromatin target of PRMT1	28875797	26	4	0	2	0	2	0	0.01	0.00	Yes			
CLPB	81570	ClpB homolog, mitochondrial AAA ATPase chaperonin	13540606	79	0	3	0	2	0	2	0.00	0.01			Yes	
CLTC	1213	clathrin heavy chain	4758012	192	2	4	1	2	1	2	0.02	0.04		51	Yes	
CNOT1	23019	CCR4-NOT transcription complex subunit 1	42716275	267	14	16	9	9	9	9	0.03	0.03		51	Yes	Yes
COPA	1314	coatomer protein complex subunit alpha	148536853	138	23	20	11	10	11	10	0.04	0.04		51	Yes	
COPB1	1315	coatomer protein complex subunit beta 1	7705369	107	4	2	4	2	4	2	0.01	0.00	Yes		Yes	
COPB2	9276	coatomer protein complex subunit beta 2	4758032	102	9	11	6	7	7	7	0.02	0.02		51	Yes	
CPSF2	53981	cleavage and polyadenylationspecific factor 2	530403998	88	3	4	3	3	3	3	0.01	0.01	Yes		Yes	
CSDE1	7812	cold shock domain containing E1	194473720	91	3	0	2	0	2	0	0.01	0.00			Yes	Yes
CSE1L	1434	chromosome segregation 1 like	29029559	110	4	8	2	4	2	4	0.01	0.02			Yes	Yes
CSNK1E	1454	casein kinase 1 epsilon	4503093	47	4	0	3	0	3	0	0.01	0.00		51		
CSTF3	1479	cleavage stimulation factor subunit 3	4557495	83	4	0	2	0	2	0	0.01	0.00				
CTNND1	1500	catenin delta 1	10835010	105	5	4	3	2	3	2	0.01	0.01			Yes	Yes
CTR9	9646	CTR9 homolog, Paf1/RNA polymerase II complex component	7661950	134	17	8	7	4	7	4	0.03	0.02			Yes	
CTSD	1509	cathepsin D	4503143	45	3	0	2	0	2	0	0.01	0.00	Yes			
DARS	1615	aspartyl-tRNA synthetase	45439306	57	11	8	5	4	5	4	0.02	0.02		51	Yes	
DCAF7	10238	DDB1 and CUL4 associated factor 7	108936958	39	8	4	4	2	4	2	0.02	0.01				
DCTN1	1639	dynactin subunit 1	13259508	127	4	5	3	3	3	3	0.01	0.01		29	Yes	Yes
DCTN2	10540	dynactin subunit 2	387527972	44	3	0	2	0	2	0	0.01	0.00	Yes	29,51	Yes	
DDB1	1642	damage specific DNA binding protein 1	148529014	127	31	29	13	10	13	10	0.06	0.06	Yes	51	Yes	
DDOST	1650	dolichyl-diphosphooligosaccharide--protein glycosyltransferase non-catalytic subunit	20070197	51	3	4	2	2	2	2	0.01	0.01		51	Yes	
DIS3	22894	DIS3 homolog, exosome endoribonuclease and 3'-5' exoribonuclease (RRP44)	190014623	109	8	9	4	5	4	5	0.02	0.02	Yes		Yes	
DNAJA1	3301	DnaJ heat shock protein family member A1	4504511	45	70	76	13	11	20	16	0.13	0.14	Yes	28,51	Yes	Yes
DNAJA2	10294	DnaJ heat shock protein family member A2	5031741	46	15	16	5	6	6	7	0.03	0.03		28,32,51	Yes	
DNAJA3	9093	DnaJ heat shock protein family member A3	205360838	52	19	22	7	5	8	6	0.04	0.04		51	Yes	
DNAJB1	3337	DnaJ heat shock protein family member B1	5453690	38	4	0	3	0	3	0	0.01	0.00			Yes	
DNAJB11	51726	DnaJ heat shock protein family member B11	7706495	41	0	6	0	4	0	4	0.00	0.01		28,51	Yes	
DNAJB6	10049	DnaJ heat shock protein family member B6	17388799	36	6	4	3	2	3	2	0.01	0.01		51	Yes	
DNAJC10	54431	DnaJ heat shock protein family member C10	24308127	91	0	5	0	3	0	3	0.00	0.01			Yes	
DNAJC7	7266	DnaJ heat shock protein family member C7	262206280	56	30	32	9	10	13	13	0.06	0.06			Yes	
DOCK7	85440	dedicator of cytokinesis 7	431822375	241	4	0	3	0	3	0	0.01	0.00		51	Yes	
DPM1	8813	dolichyl-phosphate mannosyltransferase subunit 1, catalytic	4503363	30	3	5	2	3	2	4	0.01	0.01	Yes		Yes	
DSC1	1823	desmocollin 1	13435361	100	4	0	2	0	2	0	0.01	0.00	Yes			
DSG2	1829	desmoglein 2	116534898	122	4	7	2	4	2	4	0.01	0.01			Yes	
DYNC1H1	1778	dynein cytoplasmic 1 heavy chain 1	33350932	532	38	29	23	17	23	17	0.07	0.06		29,51	Yes	Yes
DYNC1I2	1781	dynein cytoplasmic 1 intermediate chain 2	24307879	71	0	4	0	2	0	2	0.00	0.01			Yes	
EDC4	23644	enhancer of mRNA decapping 4	45827771	152	3	0	2	0	2	0	0.01	0.00			Yes	Yes
EIF3E	3646	eukaryotic translation initiation factor 3 subunit E	4503521	52	2	4	2	3	2	3	0.00	0.01				Yes
EIF3F	8665	eukaryotic translation initiation factor 3 subunit F	4503519	38	3	3	2	2	2	2	0.01	0.01		51	Yes	Yes
EIF4A1	1973	eukaryotic translation initiation factor 4A1	325197164	40	5	4	3	2	3	2	0.04	0.04		51	Yes	Yes
EIF4G1	1981	eukaryotic translation initiation factor 4 gamma 1	302699237	176	5	4	3	2	3	2	0.01	0.01		51	Yes	Yes
EMD	2010	emerin	4557553	29	7	9	4	5	4	5	0.01	0.02		51	Yes	
EPB41L2	2037	erythrocyte membrane protein band 4.1 like 2	4503579	113	5	4	3	2	3	2	0.01	0.01		51	Yes	
EPRS	2058	glutamyl-prolyl-tRNA synthetase	62241042	171	36	40	13	13	16	14	0.07	0.08		51	Yes	
FAF2	23197	Fas associated factor family member 2	24797106	53	5	0	4	0	4	0	0.01	0.00		32	Yes	
FAM83H	286077	family with sequence similarity 83 member H	157311635	127	3	0	2	0	2	0	0.01	0.00			Yes	
FAR1	84188	fatty acyl-CoA reductase 1	24308324	59	10	13	4	5	4	5	0.02	0.03		28	Yes	
FARSA	2193	phenylalanyl-tRNA synthetase alpha subunit	4758340	58	0	6	0	2	0	2	0.00	0.01		51	Yes	
FIG4	9896	FIG4 phosphoinositide 5-phosphatase	7662034	104	3	0	2	0	2	0	0.01	0.00				
FKBP5	2289	FK506 binding protein 5	4758384	51	27	18	8	4	9	6	0.05	0.03			Yes	
FKBP8	23770	FK506 binding protein 8	768001698	44	8	0	2	0	3	0	0.02	0.00		51	Yes	
FLNC	2318	filamin C	116805322	291	3	0	2	0	2	0	0.01	0.00		51	Yes	
FMR1	2332	fragile X mental retardation 1	297374779	69	2	0	2	0	2	0	0.00	0.00	Yes		Yes	

FUBP3	8939	far upstream element binding protein 3	100816392	62	4	0	2	0	2	0	0.01	0.00				Yes
FUS	2521	FUS RNA binding protein	283135173	53	0	2	0	1	0	1	0.00	0.02		51	Yes	Yes
FXR2	9513	FMR1 autosomal homolog 2	259013556	74	3	0	2	0	2	0	0.01	0.00			Yes	
G3BP1	10146	G3BP stress granule assembly factor 1	578810779	52	7	3	4	2	4	2	0.01	0.01		51	Yes	Yes
GALK1	2584	galactokinase 1	4503895	42	4	6	3	2	3	2	0.01	0.01	Yes		Yes	
GART	2618	phosphoribosylglycinamide formyltransferase, phosphoribosylglycinamide synthetase, phosphoribosylaminoimidazole synthetase	530418825	108	7	9	4	5	4	5	0.01	0.02			Yes	
GATAD2B	57459	GATA Zinc Finger Domain Containing 2B	767909989	65	3	7	3	3	3	4	0.01	0.01				
GBF1	8729	golgi brefeldin A resistant guanine nucleotide exchange factor 1	313747582	206	3	3	2	3	2	3	0.01	0.01			Yes	
GCN1	10985	GCN1, eIF2 alpha kinase activator homolog	54607053	293	37	30	18	14	19	14	0.07	0.06	Yes			
GET4	51608	golgi to ER traffic protein 4	38570062	37	3	0	2	0	2	0	0.01	0.00				
GLUD1	2746	glutamate dehydrogenase 1	4885281	61	4	3	3	2	3	2	0.01	0.01			Yes	
GTF2I	2969	general transcription factor IIi	14670350	112	55	61	18	21	18	24	0.10	0.12	Yes	51	Yes	
GTF3C1	2975	general transcription factor IIIC subunit 1	101943240	239	0	5	0	3	0	3	0.00	0.01			Yes	
GTF3C3	9330	general transcription factor IIIC subunit 3	6912398	101	12	15	4	5	4	5	0.02	0.03			Yes	
GTF3C4	9329	general transcription factor IIIC subunit 4	156119605	92	0	3	0	2	0	2	0.00	0.01			Yes	
GTF3C5	9328	general transcription factor IIIC subunit 5	170763506	60	6	5	3	3	3	3	0.01	0.01				
HACD3	51495	3-hydroxyacyl-CoA dehydratase 3	117168248	43	5	0	4	0	4	0	0.01	0.00				
HADHA	3030	hydroxyacyl-CoA dehydrogenase/3-ketoacyl-CoA thiolase/enoyl-CoA hydratase , alpha subunit	20127408	83	3	0	3	0	3	0	0.01	0.00	Yes	51	Yes	
HAT1	8520	histone acetyltransferase 1	4504341	50	0	6	0	2	0	2	0.00	0.01			Yes	
HAUS6	54801	HAUS augmin like complex subunit 6	31377562	109	0	3	0	2	0	2	0.00	0.01				
HAX1	10456	HCLS1 associated protein X-1	13435356	32	8	6	4	4	4	4	0.02	0.01		51	Yes	
HCFC1	3054	host cell factor C1	578838841	213	8	8	5	3	5	3	0.02	0.02			Yes	
HEATR1	55127	HEAT Repeat Containing 1	767912504	242	3	0	2	0	2	0	0.01	0.00			Yes	
HLA-A	3105	major histocompatibility complex, class I, A	530429121	44	5	5	2	2	2	3	0.01	0.01				
HLA-B	3106	major histocompatibility complex, class I, B	17986001	40	0	3	0	2	0	2	0.00	0.01				
HNRNPA3	220988	heterogeneous nuclear ribonucleoprotein A3	530369990	40	6	0	2	0	2	0	0.01	0.00	Yes	51	Yes	Yes
HNRNPH2	3188	heterogeneous nuclear ribonucleoprotein H2	9624998	49	4	0	2	0	2	0	0.01	0.00	Yes		Yes	Yes
HNRNPM	4670	heterogeneous nuclear ribonucleoprotein M	14141152	78	4	3	2	2	2	2	0.03	0.03	Yes	51	Yes	
HPRT1	3251	hypoxanthine phosphoribosyltransferase 1	4504483	25	6	0	4	0	4	0	0.01	0.00				
HPS6	79803	HPS6, biogenesis of lysosomal organelles complex 2 subunit 3	31881785	83	0	4	0	2	0	2	0.00	0.01			Yes	
HSD17B4	3295	hydroxysteroid 17-beta dehydrogenase 4	4504505	80	0	4	0	2	0	2	0.00	0.01			Yes	
HSPA4	3308	heat shock protein family A member 4	38327039	94	4	3	3	2	3	2	0.01	0.01		51	Yes	Yes
HSPB1	3315	heat shock protein family B member 1	4504517	23	10	9	3	3	3	3	0.09	0.08		51	Yes	Yes
HSPH1	10808	heat shock protein family H member 1	42544159	97	19	8	9	5	9	5	0.04	0.02		51	Yes	
HUWE1	10075	HECT, UBA and WWE domain containing 1, E3 ubiquitin protein ligase	530426344	482	29	20	15	8	15	8	0.05	0.04	Yes	29	Yes	
IARS	3376	isoleucyl-tRNA synthetase	94721241	145	18	22	8	7	9	7	0.03	0.04	Yes	51	Yes	
ILF2	3608	interleukin enhancer binding factor 2	24234747	43	0	4	0	2	0	2	0.00	0.04		51	Yes	
IMMT	10989	inner membrane mitochondrial protein	154354962	84	2	5	2	3	2	3	0.00	0.01		28,51	Yes	
IRS4	8471	insulin receptor substrate 4	4504733	134	10	13	5	6	6	7	0.09	0.12			Yes	
KDM1A	23028	lysine demethylase 1A	530360925	95	6	0	2	0	2	0	0.01	0.00		51	Yes	
KEAP1	9817	kelch like ECH associated protein 1	530415398	70	0	4	0	2	0	2	0.00	0.01			Yes	
KPNA2	3838	karyopherin subunit alpha 2	4504897	58	6	4	3	2	3	2	0.05	0.04			Yes	Yes
KPNB1	3837	karyopherin subunit beta 1	19923142	97	7	5	4	3	5	3	0.01	0.01	Yes	51	Yes	Yes
LARS	51520	leucyl-tRNA synthetase	108773810	134	9	14	6	8	6	8	0.02	0.03	Yes	51	Yes	
LAS1L	81887	LAS1 like, ribosome biogenesis factor	13654270	83	4	0	3	0	3	0	0.01	0.00			Yes	
LMNA	4000	lamin A/C	27436946	74	6	4	4	3	4	3	0.01	0.01	Yes		Yes	Yes
LRPPRC	10128	leucine rich pentatricopeptide repeat containing	31621305	158	10	9	7	6	8	6	0.02	0.02	Yes		Yes	
LUC7L3	51747	LUC7 like 3 pre-mRNA splicing factor	52426743	51	6	0	3	0	3	0	0.01	0.00	Yes	51	Yes	
MAGED1	9500	MAGE family member D1	52632381	92	26	33	9	9	11	11	0.05	0.06		51	Yes	Yes
MAGED2	10916	MAGE family member D2	29171705	65	5	14	2	5	2	8	0.01	0.03	Yes		Yes	Yes
MAGED4	728239	MAGE family member D4	440546419	83	7	10	4	4	4	4	0.01	0.02				
MARS	4141	methionyl-tRNA synthetase	14043022	101	10	6	3	2	3	2	0.02	0.01	Yes	51	Yes	Yes
MATR3	9782	matrin 3	62750354	95	0	4	0	2	0	3	0.00	0.04			Yes	
MCM2	4171	minichromosome maintenance complex component 2	33356547	102	0	4	0	2	0	2	0.00	0.01			Yes	
MCM5	4174	minichromosome maintenance complex component 5	23510448	82	0	4	0	3	0	3	0.00	0.04		51	Yes	Yes
MCM7	4176	minichromosome maintenance complex component 7	33469968	81	4	6	3	4	3	4	0.01	0.01			Yes	Yes
MDN1	23195	midasin AAA ATPase 1	24415404	633	10	14	7	6	7	6	0.02	0.03			Yes	
MKRN2	23609	makorin ring finger protein 2	32880199	47	0	3	0	2	0	2	0.00	0.01				
MSH6	2956	mutS homolog 6	4504191	153	5	5	2	3	2	3	0.01	0.01			Yes	Yes
MTHFD1	4522	methylenetetrahydrofolate dehydrogenase, cyclohydrolase and formyltetrahydrofolate synthetase 1	222136639	102	6	4	4	2	4	2	0.01	0.01		51	Yes	Yes
NCAPD3	23310	non-SMC Condensin II Complex Subunit D3	767969890	169	3	3	3	3	3	3	0.01	0.01			Yes	
NDUFS3	4722	NADH:ubiquinone oxidoreductase core subunit S3	4758788	30	4	6	2	2	2	2	0.01	0.01			Yes	
NEFM	4741	neurofilament, medium polypeptide	157738649	102	7	6	4	3	4	3	0.01	0.01		51	Yes	
NOP56	10528	NOP56 ribonucleoprotein	32483374	66	6	5	3	2	3	2	0.01	0.01			Yes	
NPEPPS	9520	aminopeptidase puromycin sensitive	158937236	103	8	9	4	4	4	4	0.02	0.02			Yes	
NSUN2	54888	NOP2/Sun RNA methyltransferase family member 2	301336155	82	8	0	3	0	3	0	0.02	0.00			Yes	Yes
NTPCR	84284	nucleoside-triphosphatase, cancer-related	14150100	21	19	16	5	5	8	6	0.04	0.03		28	Yes	
NUDC	10726	nuclear distribution C, dynein complex regulator	5729953	38	10	2	5	2	6	2	0.02	0.00		51		Yes
NUP133	55746	nucleoporin 133	26051235	129	10	15	8	7	8	7	0.02	0.03	Yes		Yes	
NUP153	9972	nucleoporin 153	24430146	154	0	5	0	3	0	3	0.00	0.01			Yes	
NUP155	9631	nucleoporin 155	24430149	155	4	6	3	4	3	4	0.01	0.01	Yes	51	Yes	
NUP160	23279	nucleoporin 160	54859722	162	3	4	2	3	2	3	0.01	0.01			Yes	
NUP205	23165	nucleoporin 205	57634534	228	3	4	2	3	2	3	0.01	0.01			Yes	Yes
NUP210	23225	nucleoporin 210	27477134	205	4	0	3	0	3	0	0.01	0.00			Yes	
NUP93	9688	nucleoporin 93	530424559	93	11	10	5	6	5	6	0.02	0.02	Yes		Yes	
NUP98	4928	nucleoporin 98	21264365	196	2	5	2	3	2	3	0.00	0.01			Yes	Yes
OBSL1	23363	obscurin like 1	144226847	207	0	4	0	2	0	2	0.00	0.01			Yes	
PAF1	54623	PAF1 homolog, Paf1/RNA polymerase II complex component	378744165	55	4	3	2	2	2	2	0.01	0.01			Yes	
PAICS	10606	phosphoribosylaminoimidazole carboxylase; phosphoribosylaminoimidazolesuccinocarboxamide synthase	119220559	48	3	0	2	0	2	0	0.01	0.00		51	Yes	
PARK7	11315	Parkinsonism associated deglycase	530360487	20	0	4	0	2	0	2	0.00	0.01		51		
PCBP2	5094	poly(RC) binding protein 2	14141166	38	15	11	3	2	6	5	0.03	0.02		32,51	Yes	Yes
PCNA	5111	proliferating cell nuclear antigen	4505641	29	0	4	0	2	0	2	0.00	0.01			Yes	Yes
PDCD11	22984	programmed cell death 11	767962234	208	8	5	4	3	4	3	0.02	0.01			Yes	
PDK3	5165	pyruvate dehydrogenase kinase 3	215422338	48	0	3	0	2	0	2	0.00	0.01			Yes	
PDLIM5	10611	PDZ and LIM domain 5	374092020	64	3	8	3	3	3	3	0.01	0.02				Yes
PDLIM7	9260	PDZ and LIM domain 7	11496885	50	0	4	0	3	0	3	0.00	0.01				
PDS5B	23047	PDS5 cohesin associated factor B	530402180	165	3	2	2	2	2	2	0.01	0.00				Yes
PELO	53918	pelota homolog	31880783	43	3	0	2	0	2	0	0.01	0.00		51	Yes	Yes
PFDN2	5202	prefoldin subunit 2	12408675	17	5	4	2	2	2	2	0.01	0.01		51	Yes	
PLK1	5347	polo like kinase 1	21359873	68	3	9	2	3	2	3	0.01	0.02		51		
PML	5371	promyelocytic leukemia	15451765	91	0	3	0	2	0	2	0.00	0.01			Yes	
POLD1	5424	DNA polymerase delta 1, catalytic subunit	530416735	121	0	3	0	2	0	2	0.00	0.01	Yes		Yes	
POLD3	10714	DNA polymerase delta 3, accessory subunit	38492356	51	2	3	2	2	2	2	0.00	0.01			Yes	
POLR2A	5430	RNA polymerase II subunit A	4505939	217	6	9	3	5	3	5	0.01	0.02			Yes	
PPP2R1A	5518	protein phosphatase 2 scaffold subunit Aalpha	21361399	65	7	5	2	2	3	2	0.06	0.05		51	Yes	Yes
PPP2R2A	5520	protein phosphatase 2 regulatory subunit Balpha	294832006	53	2	4	2	2	2	2	0.00	0.01		51	Yes	
PPP5C	5536	protein phosphatase 5 catalytic subunit	5453958	57	4	0	2	0	2	0	0.01	0.00			Yes	
PPP6C	5537	protein phosphatase 6 catalytic subunit	183603929	39	3	0	2	0	2	0	0.01	0.00			Yes	
PRDX6	9588	peroxiredoxin 6	4758638	25	5	7	2	3	3	4	0.01	0.01	Yes	51	Yes	Yes
PSMA1	5682	proteasome subunit alpha 1	23110935	30	7	7	3	2	3	2	0.01	0.01		51	Yes	
PSMA3	5684	proteasome subunit alpha 3	23110939	28	8	3	3	3	3	3	0.02	0.01		51	Yes	
PSMA5	5686	proteasome subunit alpha 5	23110942	26	11	6	4	3	5	3	0.02	0.01		51	Yes	
PSMA6	5687	proteasome subunit alpha 6	23110944	27	9	0	3	0	4	0	0.02	0.00	Yes	51	Yes	
PSMA7	5688	proteasome subunit alpha 7	4506189	28	17	16	6	7	6	7	0.03	0.03		51	Yes	
PSMB1	5689	proteasome subunit beta 1	4506193	26	7	7	3	5	4	5	0.01	0.01		51	Yes	
PSMB2	5690	proteasome subunit beta 2	4506195	23	5	5	3	4	3	4	0.01	0.01			Yes	
PSMB5	5693	proteasome subunit beta 5	4506201	28	6	6	3	2	3	2	0.01	0.01			Yes	
PSMB6	5694	proteasome subunit beta 6	23110925	25	5	5	2	2	2	2	0.01	0.01			Yes	
PSMC1	5700	proteasome 26S subunit, ATPase 1	24430151	49	6	6	2	2	2	2	0.01	0.01	Yes	51	Yes	
PSMC2	5701	proteasome 26S subunit, ATPase 2	4506209	49	22	18	8	7	8	7	0.04	0.03	Yes	51	Yes	
PSMC3	5702	proteasome 26S subunit, ATPase 3	21361144	49	12	9	7	5	7	5	0.02	0.02	Yes	51	Yes	
PSMC4	5704	proteasome 26S subunit, ATPase 4	24430155	44	5	0	3	0	3	0	0.01	0.00			Yes	
PSMC5	5705	proteasome 26S Subunit, ATPase 5	578831151	46	10	10	5	5	6	7	0.02	0.02		51	Yes	
PSMC6	5706	proteasome 26S subunit, ATPase 6	195539395	46	5	6	3	4	3	4	0.01	0.01	Yes	51	Yes	
PSMD1	5707	proteasome 26S subunit, non-ATPase 1	25777600	106	8	8	5	3	5	4	0.02	0.02	Yes	51	Yes	
PSMD11	5717	proteasome 26S subunit, non-ATPase 11	28872725	47	14	9	4	4	5	4	0.03	0.02		51	Yes	
PSMD12	5718	proteasome 26S subunit, non-ATPase 12	4506221	53	4	2	3	2	3	2	0.01	0.00		51	Yes	
PSMD13	5719	proteasome 26S subunit, non-ATPase 13	157502193	43	0	3	0	2	0	2	0.00	0.01		51	Yes	
PSMD14	10213	proteasome 26S subunit, non-ATPase 14	5031981	35	0	3	0	2	0	2	0.00	0.01	Yes	51	Yes	
PSMD2	5708	proteasome 26S subunit, non-ATPase 2	25777602	100	18	10	7	4	8	4	0.03	0.02		51	Yes	Yes
PSMD3	5709	proteasome 26S subunit, non-ATPase 3	25777612	61	12	8	8	6	8	6	0.02	0.02	Yes	51	Yes	

PSMD6	9861	proteasome 26S subunit, non-ATPase 6	422398832	52	9	7	4	4	5	4	0.02	0.01		51		
PSMD7	5713	proteasome 26S subunit, non-ATPase 7	25777615	37	3	0	2	0	2	0	0.01	0.00		51	Yes	
PSMD8	5714	proteasome 26S subunit, non-ATPase 8	156631005	40	0	6	0	2	0	2	0.00	0.01		29	Yes	
PSME3	10197	proteasome activator subunit 3	30410794	30	10	11	5	4	6	5	0.02	0.02		51	Yes	
PUM1	9698	pumilio RNA binding family member 1	13491166	126	3	0	2	0	2	0	0.01	0.00				Yes
QARS	5859	glutaminyl-tRNA synthetase	441478305	87	8	7	3	4	3	4	0.02	0.01		51	Yes	
RAB1B	81876	RAB1B, member RAS oncogene family	13569962	22	5	0	2	0	2	0	0.01	0.00	Yes	51		
RAD21	5885	RAD21 cohesin complex component	5453994	72	3	0	3	0	3	0	0.01	0.00				Yes
RANBP2	5903	RAN binding protein 2	150418007	358	29	25	14	10	15	10	0.05	0.05	Yes		Yes	
RANBP9	10048	RAN binding protein 9	39812378	78	4	0	2	0	2	0	0.01	0.00		51	Yes	
RANGAP1	5905	Ran GTPase activating protein 1	530420276	70	8	11	5	3	5	3	0.02	0.02		51	Yes	
RARS	5917	arginyl-tRNA synthetase	15149476	75	9	8	5	6	5	6	0.02	0.02	Yes	51	Yes	
RB1CC1	9821	RB1 inducible coiled-coil 1	134304846	183	17	13	11	8	11	8	0.03	0.03	Yes	29	Yes	
RBBP7	5931	RB binding protein 7, chromatin remodeling factor	4506439	48	3	4	2	2	2	2	0.03	0.04	Yes	51	Yes	
RBM14	10432	RNA binding motif protein 14	5454064	69	0	6	0	2	0	2	0.00	0.01		51	Yes	
RBM4	5936	RNA binding motif protein 4	93277122	40	4	6	2	3	2	3	0.01	0.01			Yes	
RCN1	5954	reticulocalbin 1	4506455	39	10	10	4	4	6	6	0.02	0.02				
RCN2	5955	reticulocalbin 2	426214088	39	12	8	6	4	6	4	0.10	0.07	Yes	51	Yes	
RFC3	5983	replication factor C subunit 3	108773789	35	5	0	2	0	2	0	0.01	0.00			Yes	Yes
RNF2	6045	ring finger protein 2	767910100	38	3	0	2	0	2	0	0.01	0.00	Yes	51		
RNF40	9810	ring finger protein 40	333440440	114	4	10	2	5	2	5	0.01	0.02				
RNGTT	8732	RNA guanylyltransferase and 5'-phosphatase	134142828	69	3	0	2	0	2	0	0.01	0.00			Yes	
RNH1	6050	ribonuclease/angiogenin inhibitor 1	767966264	50	8	0	2	0	2	0	0.02	0.00			Yes	Yes
RPN1	6184	ribophorin I	4506675	69	5	3	4	2	4	2	0.01	0.01		51	Yes	
RPRD1A	55197	regulation of nuclear pre-mRNA domain containing 1A	742670578	33	3	0	2	0	2	0	0.01	0.00				
RRBP1	6238	ribosome binding protein 1	110611220	109	0	3	0	2	0	2	0.00	0.01		51	Yes	
RUVBL2	10856	RuvB like AAA ATPase 2	5730023	51	30	25	10	8	11	9	0.06	0.05		51	Yes	
SAMHD1	25939	SAM and HD domain containing deoxynucleoside triphosphate triphosphohydrolase 1	38016914	72	0	4	0	2	0	2	0.00	0.01			Yes	
SDF4	51150	stromal cell derived factor 4	18699732	42	4	7	3	5	3	5	0.01	0.01			Yes	
SEC16A	9919	SEC16 homolog A, endoplasmic reticulum export factor	530426853	254	14	12	8	5	8	5	0.03	0.02			Yes	
SEC24C	9632	SEC24 homolog C, COPII coat complex component	38373669	118	4	3	3	2	3	2	0.01	0.01			Yes	Yes
SEC61B	10952	Sec61 translocon beta subunit	5803165	10	6	0	2	0	3	0	0.01	0.00		51	Yes	
SERPINB3	6317	serpin family B member 3	5902072	45	3	0	2	0	2	0	0.01	0.00	Yes			
SERPINH1	871	serpin Family H Member 1	767968948	46	11	0	6	0	6	0	0.02	0.00			Yes	
SFXN1	94081	sideroflexin 1	23618867	36	4	5	2	2	2	2	0.01	0.01	Yes			
SFXN4	119559	sideroflexin 4	47458811	38	8	5	2	2	3	2	0.02	0.01			Yes	
SLC16A1	6566	solute carrier family 16 member 1	767905752	?	0	5	0	2	0	3	0.00	0.01		51	Yes	
SLC25A1	6576	solute carrier family 25 member 1	21389315	34	0	5	0	2	0	2	0.00	0.01			Yes	
SLC25A10	1468	solute carrier family 25 member 10	20149598	31	5	7	3	2	3	3	0.01	0.01			Yes	
SLC25A11	8402	solute carrier family 25 member 11	21361114	34	14	28	6	6	7	8	0.03	0.05	Yes	28,32	Yes	
SLC25A22	79751	solute carrier family 25 member 22	767966553	34	13	2	4	2	4	2	0.02	0.00			Yes	
SLC25A3	5250	solute carrier family 25 member 3	47132595	40	22	32	5	8	8	13	0.04	0.06	Yes	28,51	Yes	
SLC25A6	293	solute carrier family 25 member 6	156071462	33	12	10	2	2	3	2	0.02	0.02	Yes	32	Yes	
SLC27A4	10999	solute carrier family 27 member 4	40807357	72	4	3	2	2	2	2	0.01	0.01			Yes	
SLC3A2	6520	solute carrier family 3 member 2	61744477	68	4	8	3	5	3	5	0.01	0.02		51	Yes	
SMARCA4	6597	SWI/SNF related, matrix associated, actin dependent regulator of chromatin, subfamily a, member 4	21071056	185	8	17	5	7	5	7	0.02	0.03			Yes	
SMARCA5	8467	SWI/SNF related, matrix associated, actin dependent regulator of chromatin, subfamily a, member 5	325651836	122	3	2	2	2	2	2	0.01	0.00				
SMC1A	8243	structural maintenance of chromosomes 1A	30581135	143	15	15	9	6	9	7	0.03	0.03	Yes		Yes	
SMC2	10592	structural maintenance of chromosomes 2	578816998	136	9	6	5	4	5	4	0.02	0.01		51	Yes	
SMC3	9126	structural maintenance of chromosomes 3	4885399	142	12	8	6	4	7	4	0.02	0.02			Yes	
SMCR8	140775	Smith-Magenis syndrome chromosome region, candidate 8	146260268	105	1135	1182	52	49	130	124	2.10	2.20	Yes	28,29,31,32	Yes	
SND1	27044	staphylococcal nuclease and tudor domain containing 1	77404397	102	5	7	4	3	4	3	0.01	0.01			Yes	Yes
SNRPE	6635	small nuclear ribonucleoprotein polypeptide E	4507129	11	3	0	2	0	2	0	0.01	0.00	Yes		Yes	
SPG20	23111	spastic paraplegia 20	530402212	73	3	0	2	0	2	0	0.01	0.00			Yes	
SPTLC1	10558	serine palmitoyltransferase long chain base subunit 1	5454084	53	0	3	0	2	0	2	0.00	0.03			Yes	
SQSTM1	8878	sequestosome 1	214830438	39	4	3	2	2	2	2	0.01	0.01		29,51		Yes
SSBP1	6742	single stranded DNA binding protein 1	374671775	17	5	0	2	0	2	0	0.01	0.00			Yes	
SSR4	6748	signal sequence receptor subunit 4	325301072	20	2	3	1	2	1	2	0.02	0.03			Yes	
SSRP1	6749	structure specific recognition protein 1	4507241	81	2	7	2	4	2	4	0.00	0.01	Yes			
STIP1	10963	stress induced phosphoprotein 1	544063423	68	37	22	10	7	14	9	0.07	0.04		51	Yes	Yes
STOML2	30968	stomatin like 2	559098406	33	2	7	2	2	2	2	0.00	0.01	Yes		Yes	
STUB1	10273	STIP1 homology and U-box containing protein 1	56181387	35	30	31	8	7	11	10	0.06	0.06		29,51	Yes	
SUGP2	10147	SURP and G-patch domain containing 2	224282117	120	0	7	0	3	0	3	0.00	0.01				Yes
SUGT1	10910	SGT1 homolog, MIS12 kinetochore complex assembly cochaperone	195963398	41	20	13	6	6	7	7	0.04	0.03	Yes		Yes	
SUPT5H	6829	SPT5 homolog, DSIF elongation factor subunit	530417099	121	3	5	2	2	2	3	0.01	0.01			Yes	
TARDBP	23435	TAR DNA binding protein	6678271	45	6	0	3	0	3	0	0.01	0.00		5,51	Yes	
TARS	6897	threonyl-tRNA synthetase	38202255	83	0	4	0	3	0	3	0.00	0.01			Yes	
TARS2	80222	threonyl-tRNA synthetase 2, mitochondrial	20070344	81	6	4	3	2	3	2	0.01	0.01			Yes	
TBC1D4	9882	TBC1 domain family member 4	114688046	147	0	3	0	2	0	2	0.00	0.01			Yes	
TCOF1	6949	treacle ribosome biogenesis factor 1	207113160	152	3	5	2	3	2	3	0.01	0.01		51	Yes	
TECR	9524	trans-2,3-enoyl-CoA reductase	24475816	36	11	15	4	3	5	4	0.02	0.03	Yes	51	Yes	
TELO2	9894	telomere maintenance 2	225545550	92	6	7	5	5	5	5	0.01	0.01			Yes	
TFRC	7037	transferrin receptor	189458817	85	4	0	2	0	2	0	0.01	0.00		51	Yes	
THOC2	57187	THO complex 2	125656165	183	9	6	4	3	4	3	0.02	0.01			Yes	
THOC3	84321	THO complex 3	14150171	39	0	4	0	3	0	3	0.00	0.01	Yes			
TIMM50	92609	translocase of inner mitochondrial membrane 50	48526509	50	8	12	3	5	4	5	0.02	0.02	Yes	51	Yes	
TMEM33	55161	transmembrane protein 33	530376690	28	8	10	4	4	4	4	0.02	0.02			Yes	
TNPO1	3842	transportin 1	133925811	102	3	5	2	3	2	3	0.01	0.01			Yes	Yes
TOP2A	7153	topoisomerase II alpha	19913406	174	5	4	3	2	3	2	0.01	0.01				
TRAFD1	10906	TRAF-type zinc finger domain containing 1	5729828	65	6	9	2	4	2	4	0.01	0.02				
TTC4	7268	tetratricopeptide repeat domain 4	156627581	45	3	0	2	0	2	0	0.01	0.00			Yes	
TUBB3	10381	tubulin beta 3 class III	50592996	50	9	13	2	2	3	3	0.02	0.03		51	Yes	Yes
TUBB4A	10382	tubulin beta 4A class IVa	574584816	50	11	10	3	4	3	4	0.02	0.02		28,51		
TUBB6	84617	tubulin beta 6 class V	14210536	50	9	18	3	4	5	6	0.02	0.03		51	Yes	
TXN	7295	thioredoxin	50592994	12	6	0	2	0	2	0	0.01	0.00	Yes	51	Yes	Yes
TXNDC5	81567	thioredoxin domain containing 5	224493972	36	5	2	4	2	4	2	0.01	0.00			Yes	
U2AF2	11338	U2 small nuclear RNA auxiliary factor 2	578833896	34	3	0	2	0	2	0	0.01	0.00	Yes	51	Yes	
UBR5	51366	ubiquitin protein ligase E3 component n-recognin 5	15147337	309	3	12	2	7	2	7	0.01	0.02			Yes	
UMPS	7372	uridine monophosphate synthetase	4507835	52	3	0	2	0	2	0	0.01	0.00			Yes	
UNC45A	55898	unc-45 myosin chaperone A	89179321	102	2	5	2	4	2	4	0.00	0.01		51	Yes	
USP15	9958	ubiquitin specific peptidase 15	14149627	109	3	0	2	0	2	0	0.01	0.00				
USP7	7874	ubiquitin specific peptidase 7	150378533	128	4	9	4	6	4	7	0.01	0.02			Yes	
USP9X	8239	ubiquitin specific peptidase 9, X-linked	145309309	292	4	5	2	3	2	3	0.01	0.01			Yes	
WDR41	55255	WD repeat domain 41	42716287	52	32	33	9	10	11	12	0.06	0.06	Yes	28,29,31,32	Yes	
WDR5	11091	WD repeat domain 5	530427102	37	0	5	0	2	0	2	0.00	0.01		51	Yes	
WDR6	11180	WD repeat domain 6	197927448	125	7	10	4	5	5	6	0.01	0.02			Yes	
WNK1	65125	WNK lysine deficient protein kinase 1	296939600	280	0	3	0	2	0	2	0.00	0.01				
XPO1	7514	exportin 1	530368070	122	13	13	7	8	8	9	0.02	0.03			Yes	
XPOT	11260	exportin for tRNA	8051636	110	0	4	0	2	0	2	0.00	0.01			Yes	
XRN1	54464	5'-3' exoribonuclease 1	110624792	194	3	0	2	0	2	0	0.01	0.00				Yes
YME1L1	10730	YME1 like 1 ATPase	21327685	86	0	4	0	3	0	3	0.00	0.01			Yes	
YTHDF2	51441	YTH N6-methyladenosine RNA binding protein 2	290542345	62	11	18	5	4	5	5	0.02	0.03			Yes	Yes
YWHAZ	7534	tyrosine 3-monooxygenase/tryptophan 5-monooxygenase activation protein zeta	530389317	28	4	9	2	2	2	2	0.01	0.02		51	Yes	
ZC3H14	79882	zinc finger CCCH-type containing 14	231570121	83	0	5	0	2	0	2	0.00	0.01				Yes
ZFR	51663	zinc finger RNA binding protein	34101286	117	0	8	0	4	0	4	0.00	0.02			Yes	
ZG16B	124220	zymogen granule protein 16B	94536866	23	6	0	2	0	2	0	0.01	0.00				
ZMYM4	9202	zinc finger MYM-type containing 4	44890068	173	5	6	3	4	3	4	0.01	0.01			Yes	
ZNF281	23528	zinc finger protein 281	526253066	93	10	7	5	4	5	4	0.02	0.01				
ZNF598	90850	zinc finger protein 598	409264581	99	0	4	0	2	0	2	0.00	0.01				

Table S3. Ubiquitinated SMCR8 lysine residues determined by MS sequencing, prediction algorithms, and the comparative phylogenetic analyses of Fig. S3

MS results	Ubiquitination prediction algorithms			Conserved in ≥8 species of Fig. S3
	UBPRED	BDM-PUB score (threshold 0.3)	UbiSite	
K189		0.63	0.69 (High)	✓
K198		0.85		✓
K222		0.58		✓
K232	MED	1.14	0.50 (High)	✓
K241	LOW			✓
K419	MED		0.51 (High)	✓
K479	HIGH	1.22		✓
K712		1.37	0.59 (High)	
K815				✓

Table S4. Post-mortem brain motor cortex tissue samples used for the analyses of Fig. 5b,c.

Source	Patient #	Type	Age at Onset	Age at Death	Sex	Race	Disease duration (months)	PMI (hours)	ImageJ value	Notes / Cause of Death
UCSD	19	CONT	N/A	80	F	W	N/A	2.5	134.8	Parkinson's disease
UCSD	23	CONT	N/A	77	M	W	N/A	6	101.5	Cardiac failure
UCSD	26	CONT	N/A	49	M	W	N/A	4	140.3	Cancer / Obesity
UCSD	31	CONT	N/A	67	M	W	N/A	3.5	396.4	Lung cancer
UCSD	39	CONT	N/A	77	M	W	N/A	2	257.3	Aortic dissection / Multiple system failure
JHU	60	CONT	N/A	80	M	W	N/A	15	192.7	Lewy body dementia / Peripheral neuropathy
UCSD	73	CONT	N/A	74	M	W	N/A	5.5	267.0	Cardiovascular failure
GBB	13-06	CONT	N/A	72	M	W	N/A	7	282.4	Diffuse alveolar damage / Pneumonia
GBB	14-06	CONT	N/A	71	F	unknown	N/A	5	302.7	Hypertensive vasculopathy, Lacunar infarcts
GBB	15-09	CONT	N/A	70	F	unknown	N/A	32	32.8	Indeterminate
CU	16	C9ALS	58	59	M	W	12	18.8	17.3	ALS
CU	25	C9ALS	68	70	M	unknown	24	19	144.1	Unknown
CU	26	C9ALS	64	68	F	W	48	13	69.2	Respiratory failure / end-stage ALS / aspiration pneumonia
GWF	15-09	C9ALS	unknown	61	F	W	unknown	5.5	15.5	Unknown
GWF	17-24	C9ALS	46	51	F	W	65	7	43.5	
GWF	14-04	C9ALS	63	67	M	W	48	4	25.1	Respiratory Failure
JHU	19	C9ALS	~48	52	M	W	36	17	4.7	
JHU	38	C9ALS	33	34	F	W	17	5	105.7	Unknown
JHU	52	C9ALS	53	56	M	W	30	13	17.9	Unknown
JHU	88	C9ALS	57	59	M	W	~48	10	0.8	
JHU	92	C9ALS	70	92	M	W	~264	9.5	1.7	

CU: Columbia University
GBB: Georgetown University
GWF: Barrow Neurological Institute
JHU: Johns Hopkins University School of Medicine
UCSD: University of California, San Diego
N/A: not applicable
